# Lysosomal trapping of palbociclib and its functional implications

**DOI:** 10.1038/s41388-019-0695-8

**Published:** 2019-01-28

**Authors:** Susana Llanos, Diego Megias, Carmen Blanco-Aparicio, Elena Hernández-Encinas, Miguel Rovira, Federico Pietrocola, Manuel Serrano

**Affiliations:** 1 Spanish National Cancer Research Center (CNIO), Madrid, Spain; 2 Institute for Research in Biomedicine (IRB Barcelona), Barcelona Institute of Science and Technology (BIST), Barcelona, Spain; 3 Catalan Institution for Research and Advanced Studies (ICREA), Barcelona, Spain

**Keywords:** Lysosomes, Senescence

## Abstract

Palbociclib is a selective inhibitor of cyclin-dependent kinases 4 and 6 (CDK4/6) approved for the treatment of some cancers. The main mechanism of action of palbociclib is to induce cell cycle arrest and senescence on responsive cells. Here, we report that palbociclib concentrates in intracellular acidic vesicles, where it can be readily observed due to its intrinsic fluorescence, and it is released from these vesicles upon dilution or washing out of the extracellular medium. This reversible storage of drugs into acidic vesicles is generally known as lysosomal trapping and, based on this, we uncover novel properties of palbociclib. In particular, a short exposure of cells to palbociclib is sufficient to produce a stable cell-cycle arrest and long-term senescence. Moreover, after washing out the drug, palbociclib-treated cells release the drug to the medium and this conditioned medium is active on susceptible cells. Interestingly, cancer cells resistant to palbociclib also accumulate and release the drug producing paracrine senescence on susceptible cells. Finally, other lysosomotropic drugs, such as chloroquine, interfere with the accumulation of palbociclib into lysosomes, thereby reducing the minimal dose of palbociclib required for cell-cycle arrest and senescence. In summary, lysosomal trapping explains the prolonged temporal activity of palbociclib, the paracrine activity of exposed cells, and the cooperation with lysosomotropic drugs. These are important features that may help to improve the therapeutic dosing and efficacy of palbociclib. Finally, two other clinically approved CDK4/6 inhibitors, ribociclib and abemaciclib, present a similar behavior as palbociclib, suggesting that lysosomal trapping is a property common to all three clinically-approved CDK4/6 inhibitors.

## Introduction

The uncontrolled cellular proliferation of tumor cells often depends on the activity of the related kinases CDK4 and CDK6, and thus these kinases are targets for anti-tumoral drugs. Palbociclib (also known as PD-0332991) is a highly selective CDK4/6 inhibitor approved for the treatment of advanced or metastasized estrogen receptor (ER)-positive and epidermal growth factor receptor 2 (HER2)-negative breast cancer in combination with aromatase inhibitors or with ER inhibitors [1, 2]. The potential of palbociclib as monotherapy or combined therapy in other types of cancers is currently being evaluated in numerous studies and clinical trials with promising results [2]. The best understood effect of palbociclib on susceptible cells is the induction of a durable cell-cycle arrest that often evolves into cellular senescence [3–6]. The inhibition of CDK4/6 results in the accumulation of the unphosphorylated form of the RB1 tumor suppressor, and this is the main mechanism responsible for cell proliferation arrest and for the global epigenetic reconfiguration of chromatin characteristic of senescent cells [7, 8]. Other effects of palbociclib are related to newly discovered functions of CDK4/6. In this regard, inhibition of CDK4/6 in cancer cells reduces the levels of the anti-oxidants NADPH and glutathione [9], stabilizes the immunosuppressive ligand PD-L1 (ref. [10]), stimulates tumor antigen presentation [10], and hyperactivates proteasomal function [11].

Cellular senescence is a stable cell cycle arrest induced in response to multiple cellular stresses and chemotherapeutic drugs [12], including CDK4/6 inhibitors [3]. Many cancer cells retain the ability to undergo senescence and, therefore, activation of senescence is a promising novel approach for cancer intervention [13, 14]. Cellular senescence is characterized by prominent phenotypic changes, such as enlarged cellular size and an augmented lysosomal compartment [15]. The latter can be visualized by staining cells for lysosomal β-galactosidase activity, which constitutes the basis for the senescence-associated β-galactosidase (SA-βgal) activity [16]. In addition to β-galactosidase, senescent cells present high levels of most tested lysosomal hydrolases [17]. The high lysosomal content of senescent cells may contribute to their potent secretory activity, generally known as senescence-associated secretory phenotype (SASP) [18], but not to the cell-cycle arrest. This is illustrated by the fact that inhibition of mTORC1 in senescent cells simultaneously reduces their SASP and their SA-βgal activity without affecting proliferation arrest [19–21].

Some drugs are membrane permeable at neutral pH when they are non-protonated, but become membrane impermeable upon protonation at acidic pH. These drugs accumulate into lysosomes and other cellular acidic compartments, a process known as lysosomal trapping. The degree of lysosomal entrapment of a molecule depends on its pKa and, for example, molecules with a pKa value of ~8 can theoretically achieve a thousand-fold higher concentration in lysosomes relative to the cytoplasm and the extracellular space [22, 23]. Of relevance, the lysosomal entrapment of these drugs is reversible since both forms of the drug, protonated (membrane impermeable) and non-protonated (membrane permeable), establish an equilibrium across cellular membranes. Accordingly, upon dilution of the drug from the external medium, lysosomal-trapped drugs diffuse out of lysosomes until a new equilibrium is reached [22]. Palbociclib, due to its pKa value of 8.64, can conceivably undergo lysosomal entrapment. In this work, we demonstrate that palbociclib is indeed reversibly sequestered into acidic vesicles and analyze the functional consequences of this feature.

## Results

### Palbociclib is reversibly stored into acidic vesicles

**Fig. 1:**
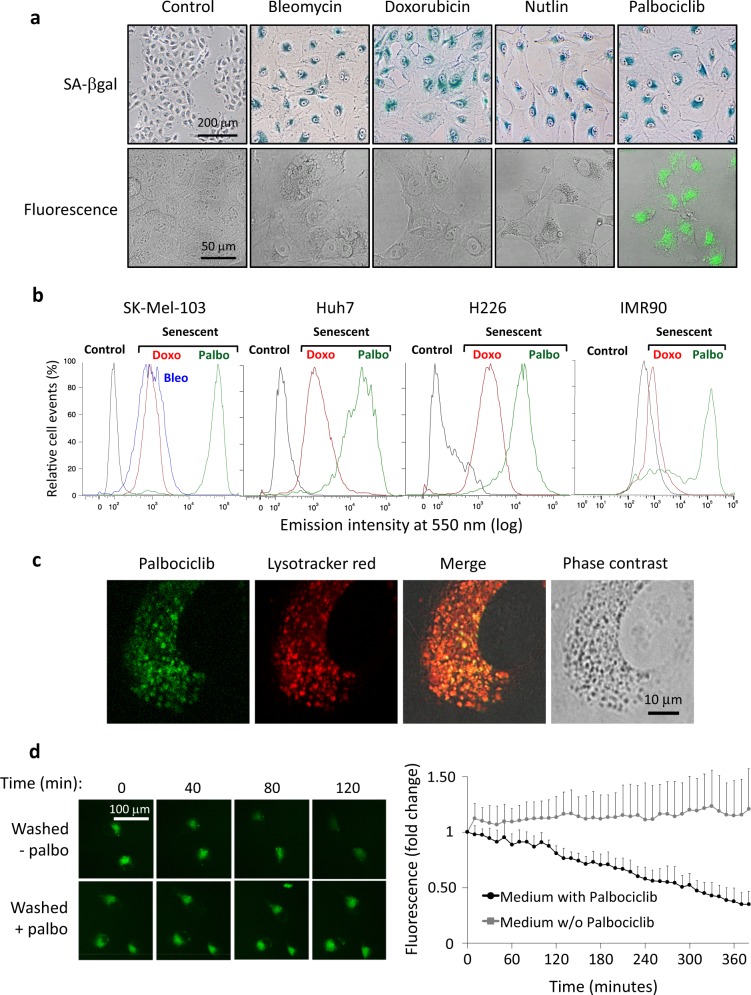
Palbociclib is reversibly stored into acidic vesicles. **a** (Top images) SA-βgal staining of SK-Mel-103 cells treated with different senescence-inducing stimuli (bleomycin 12 mUnits/ml, doxorubicin 10 nM, nutlin 10 µM, palbociclib 1 µM) for 7 days. The drugs were added only once and the media were not changed for the length of the treatment. (Bottom images) Confocal images of live control and senescent cells (excitation 405 mm/emission 500–550 nm). **b** Representative flow cytometry histogram plots of the indicated cells after treatment with bleomycin (12 mUnits/ml), doxorubicin (10 nM for cancer cells or 250 nM for IMR90 cells), and palbociclib (5 μM) during 7 days. Cells were excited with a 405 nm violet laser and the intensity of the emitted fluorescence was detected at 550 nm. **c** Confocal images illustrating the co-localization of lysotracker red and palbociclib-fluorescence in SK-Mel-103 cells treated with palbociclib 1 µM for 7 days. To improve palbociclib detection cells were incubated with 4 µM palbociclib 1 h prior to image acquisition. **d** Analysis of palbociclib-fluorescence in live SK-Mel-103 cells treated with palbociclib 1 µM for 7 days and then seeded in flow chamber slides in the presence 4 µM palbociclib. 24 h after seeding, chamber slides were flowed with medium containing or not palbociclib 4 µM and pictures were taken every 10 min. The graph represents the average and standard deviation of palbociclib-fluorescence in 10 individual cells normalized to time 0

To study the subcellular localization of palbociclib, we used the human melanoma cell line SK-Mel-103, which is RB1- and p53-functional [24, 25]. These cells efficiently underwent senescence upon treatment with DNA-damaging drugs, such as bleomycin or doxorubicin, the MDM2 inhibitor nutlin, or palbociclib, as revealed by their increased cellular size and augmented SA-βgal activity in essentially all the cells of the cultures (Fig. 1a). As expected, this was accompanied by loss of phospho-RB1 and FOXM1 (Figure S1a). FOXM1 is a well-established phosphorylation target of CDK4/6 that is highly unstable in the absence of CDK4/6 activity [26]. The secretion of inflammatory cytokines further confirmed the induction of senescence (Figure S1b). Of note, we used 1 µM palbociclib, which is standard in many cellular studies [3] and which is not far from the concentration range achieved in the plasma of patients (0.2–0.4 µM; [27, 28]).

We observed that palbociclib, when excited with 405 nm light, emits a strong fluorescence signal at ~500 nm, both at neutral (7.5) and acidic (4.5) pH (Figure S1c). In live cells, it was also possible to detect palbociclib fluorescence using the same excitation laser (405 nm) and collecting the emitted light using a 500–550 nm filter; under these conditions, palbociclib-senescent cells presented a strong fluorescence signal that was absent in senescent cells induced with bleomycin, doxorubicin, or nutlin (Fig. 1a). The signal produced by palbociclib-senescent cells was detectable by FACS as a peak of higher intensity than the peak corresponding to the autofluorescence characteristic of senescent cells induced by other means, such as doxorubicin or bleomycin (Fig. 1b). This finding was observed in all the tested palbociclib-responsive cells, including melanoma cells (SK-Mel-103), liver cancer cells (HuH7), lung cancer cells (H226), and normal human fibroblasts (IMR90) (Fig. 1b). By microscopy, palbociclib fluorescence presented a spotted perinuclear localization that was reminiscent of the localization of lysosomal SA-βgal activity (Fig. 1a). Indeed, palbociclib fluorescence co-localized with lysotracker, a fluorescent dye used for labeling acidic organelles in live cells (Fig. 1c). These observations strongly suggest that palbociclib accumulates into lysosomes though they do not rule out its presence in other cellular locations albeit at lower concentrations.

To demonstrate that palbociclib can diffuse out of the lysosomes upon washing out the drug from the extracellular medium, we quantified palbociclib fluorescence in live senescent SK-Mel-103 cells (treated with 1 µM palbociclib for 7 days) seeded in flow chamber slides. To improve detection, cells were exposed to an additional treatment of 4 µM palbociclib for 24 h. After this, cells were flowed with drug-free medium and confocal images, taken every 10 minutes, were quantified. This experimental setting showed that the fluorescent signal of palbociclib-senescent cells faded away when flowed with drug-free medium (Fig. 1d). It took about ~4.5 h to observe a reduction of ~50% in the intracellular palbociclib fluorescence. The same kinetic was observed when images were taken at 1 h intervals, thus indicating that the decrease in the fluorescent signal is not due to laser photo-bleaching (Figure S1d). Osteosarcoma Saos2 cells harbor a homozygous deletion in the *RB1* gene [29] and are therefore resistant to palbociclib in the sense that they do not undergo neither cell-cycle arrest nor senescence (Figure S1e to g). Interestingly, Saos2 cells treated with palbociclib also exhibited a fluorescent signal with the same pattern as lysosomes, albeit palbociclib-fluorescence was of lower intensity compared to senescent SK-Mel-103 cells (Figure S1h). Palbociclib intracellular fluorescence was washed out more rapidly from Saos2 cells (~50% in ~1 h) (Figure S1i) than from palbociclib-senescent SK-Mel-103 cells (Fig. 1d). We also followed the kinetics of palbociclib uptake in senescent SK-Mel-103 cells. For this, cells that had been rendered senescent with 1 µM palbociclib for 7 days were flowed with media containing 4 μM palbociclib. The increase in fluorescence was readily detected and reached a plateau after ~3 h (Figure S1j). Taken together, these observations are consistent with the reversible entrapment of palbociclib into lysosomes, a process known as lysosomal trapping. This phenomenon occurs both in senescent and in non-senescent cells, although the amount of palbociclib trapped in senescent cells is higher than in non-senescent cells, probably due to the characteristic larger size of the lysosomal compartment of senescent cells.

### Short- and long-term effects of palbociclib on lysosomal function

**Fig. 2:**
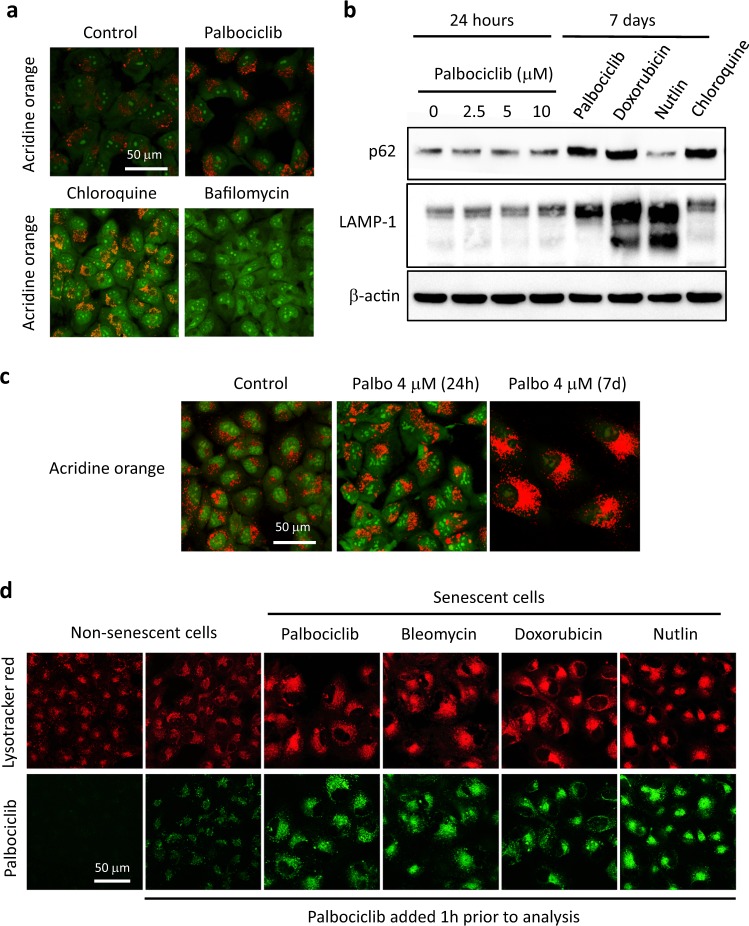
Short- and long-term effects of palbociclib on lysosomal function. **a** Confocal images of acridine orange-stained SK-Mel-103 after 1 h treatment with the indicated compounds (palbociclib 4 µM, chloroquine 50 µM, bafilomycin 40 nM). **b** Western blot depicting the levels of the autophagy marker p62 and the lysosomal marker LAMP-1 in SK-Mel-103 cells treated with the indicated concentrations of palbociclib for 24 h, or with the indicated compounds (palbociclib 1 µM, doxorubicin 10 nM, nutlin 10 µM) for 7 days. All the drugs were added once and the media were not changed for the duration of the treatment. Lysates from cells treated with 5 µM chloroquine for 48 h were included as control for autophagy inhibition. **c** Confocal images of acridine orange signal in control and palbociclib-treated SK-Mel-103 cells. **d** Palbociclib-fluorescence signal in non-senescent and senescent cells: SK-Mel-103 cells were treated for 7 days with the indicated senescence-inducing drugs (palbociclib 1 µM, bleomycin 12 mUnits/ml, doxorubicin 10 nM, nutlin 10 µM). The drugs were added only once and the culture media were not changed for the length of the treatment. Subsequently, control (non-senescent) and senescent cells were incubated in the absence (untreated) or presence of 4 μM palbociclib and lysotracker red for 1 h prior to confocal microscopy

The accumulation of basic molecules within lysosomes may elevate their pH and this may interfere with lysosomal function [23]. To assess the short-term effect of palbociclib on the lysosomal compartment, we stained cells with acridine orange (AO). AO is a fluorescent dye whose emission spectrum changes depending on the pH: emitting a red signal at acidic pH, such as within functional lysosomes, and a green signal at neutral pH, such as in the cytosol and nucleus where it preferentially stains nucleoli [27]. As expected, AO produced a red perinuclear spotted signal and a weak green cytosolic fluorescence in normal SK-Mel-103 cells (Fig. 2a). As additional controls, we used two drugs often employed to produce lysosomal basification, namely, chloroquine and bafilomycin A1. Upon treatment with chloroquine, the perinuclear compartment became orange, indicative of moderate lysosome basification, and the cytosol produced a more intense green signal. When cells were incubated with bafilomycin A1, which results in strong lysosomal basification, AO produced a homogeneous pan-cytoplasmic green signal that included the perinuclear region (Fig. 2a). In contrast to chloroquine or bafilomycin A1, treatment with palbociclib for the same period of time (1 h) did not affect the fluorescent pattern of AO, even when palbociclib was used at high concentrations (4 μM), thereby indicating that palbociclib does not detectably alter the lysosomal pH, even when used at doses above therapeutic levels (Fig. 2a).

To further assess lysosomal function, we measured the levels of LAMP-1 and p62. LAMP-1 is a glycoprotein associated to the lysosomal membrane, generally used as an indicator of lysosomal content; p62 is an autophagosome cargo protein that is degraded upon fusion of autophagosomes with lysosomes and, therefore, p62 levels are inversely correlated with the autophagic flux [28]. Upon short-time treatment with palbociclib (24 h), these markers did not undergo significant changes (Fig. 2b). However, after a longer incubation period (7 days), when cells underwent senescence, a significant elevation on the levels of both proteins was observed in palbociclib-treated cells suggesting an increase in lysosomal content and an impaired autophagic flux (Fig. 2b). Importantly, however, these changes were also observed upon treatment with other senescence-inducing stimuli (Fig. 2b). Therefore, increased lysosomal content and impaired autophagic flux are changes associated to multiple types of senescence and are not unique to palbociclib-induced senescence.

The expansion of the lysosomal compartment characteristic of senescent SK-Mel-103 cells was evident upon staining with acridine orange (Fig. 2c) or with lysotracker (Fig. 2d). Since palbociclib is trapped in lysosomes and the lysosomal compartment is greatly expanded upon senescence, we hypothesized that senescent cells may accumulate more palbociclib than control cells. Indeed, senescent cells, induced in several different ways, when briefly exposed to palbociclib (1 h prior to analysis), presented much higher levels of palbociclib-fluorescence than non-senescent cells similarly exposed to palbociclib (Fig. 2d). This probably reflects the larger size of the lysosomal compartment characteristic of senescent cells.

A common practice when treating cultured cells with chemical compounds is to refresh the culture medium every other day with drug-containing medium to prevent the decline of the drug. In the case of palbociclib, this practice resulted in a dramatic progressive accumulation of palbociclib into lysosomes in SK-Mel-103 cells together with a parallel expansion of the lysosomal compartment (Figure S2a,b). Interestingly, successive re-additions of palbociclib did not affect the amount of palbociclib in Saos2 cells and did not produce expansion of the lysosomal compartment (Figure S2a). This suggests that, in contrast to non-senescent cells, the size of the lysosomal compartment of senescent cells can be expanded according to the exposure to palbociclib.

### Lysosomal trapping contributes to the long-term effects palbociclib

**Fig. 3:**
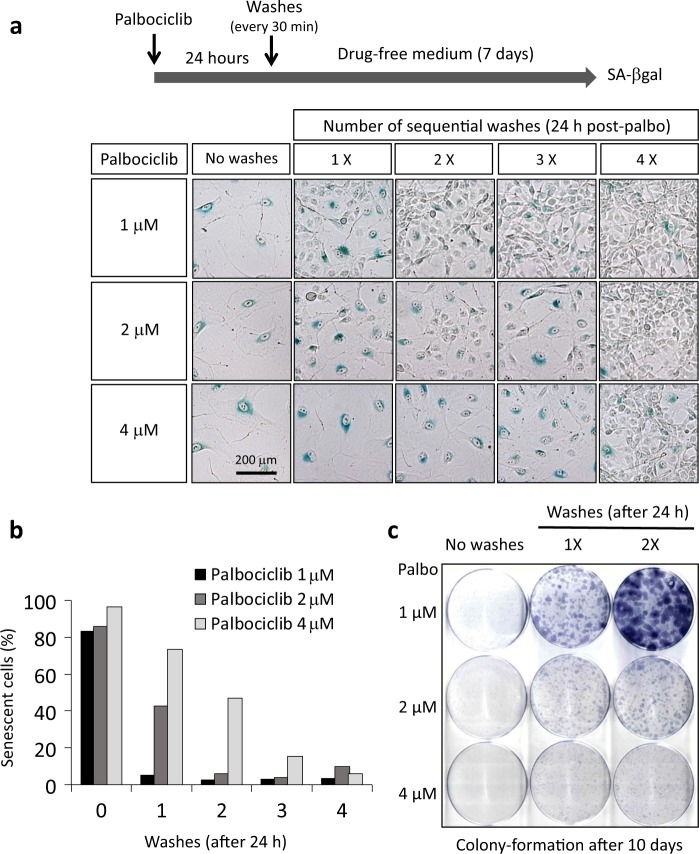
Lysosomal trapping contributes to the long-term effects palbociclib. **a**, **b** SK-Mel-103 cells were treated with different concentrations of palbociclib for 24 h. Following palbociclib treatment, cells were washed with PBS and added fresh media lacking palbociclib. This procedure was repeated for the indicated number of times. Between successive changes of media, cultures were resting for 30 min. **a** The SA-βgal activity of the cultures was assessed 7 days after palbociclib withdrawal. **b** The percentage of senescent cells was quantified 7 days after palbociclib withdrawal by flow cytometry. Control and senescent cells were differentiated on the basis of their scatter patterns (FCS-A vs SSC-A) (see Figures S3b,c). A total of two independent experiments were performed, one is shown in this panel and the other one is shown with the FACS profiles in the Supplementary Information (Figure S3d). **c** SK-Mel-103 cells (1000 cells) were seeded in 35 mm dishes and treated with the indicated concentrations of palbociclib. 24 h later, cells were washed, as in **a**, for the specified number of times. Colonies were visualized by crystal violet staining 10 days after palbociclib withdrawal

Cellular senescence is a slow process that generally requires a period of about 7 days [30, 31]. We wondered whether exposure of cells to palbociclib for just 24 h could be sufficient to trigger senescence. We first confirmed that 24 h treatment produced proliferative arrest, as expected (Figure S3a). Cultures were then washed with drug-free medium from 1 to 4 times, at 30 min intervals. After these serial washes, cells were incubated for 7 additional days without media changes and senescence was assessed visually by SA-βgal staining (Fig. 3a). Interestingly, despite the short time of exposure (24 h), a fraction of cells underwent senescence in a proportion that was dependent on the initial dose of palbociclib and on the number of washes. These effects were quantified by FACS (FCS-A vs. SSC-A) (Fig. 3b and S3d) taking advantage of the dramatic changes in size and granularity of senescent SK-Mel-103 cells (Figures S3b to d). In a similar series of experiments, cells were incubated with palbociclib for a long period of time (7 days) and then subjected to serial washes. In this case, cells remained largely senescent after extensive media washes proving the stability of the senescent phenotype (Figure S3e). We conclude that a short period (24 h) of exposure to palbociclib can be sufficient to induce long-term senescence at least in a fraction of cells. The storage and subsequent release of palbociclib from lysosomes may increase the time of exposure of cells to the drug contributing to the sustained inhibition of CDK4/6 and the induction of senescence.

### Paracrine senescence by release of lysosome-trapped palbociclib

**Fig. 4:**
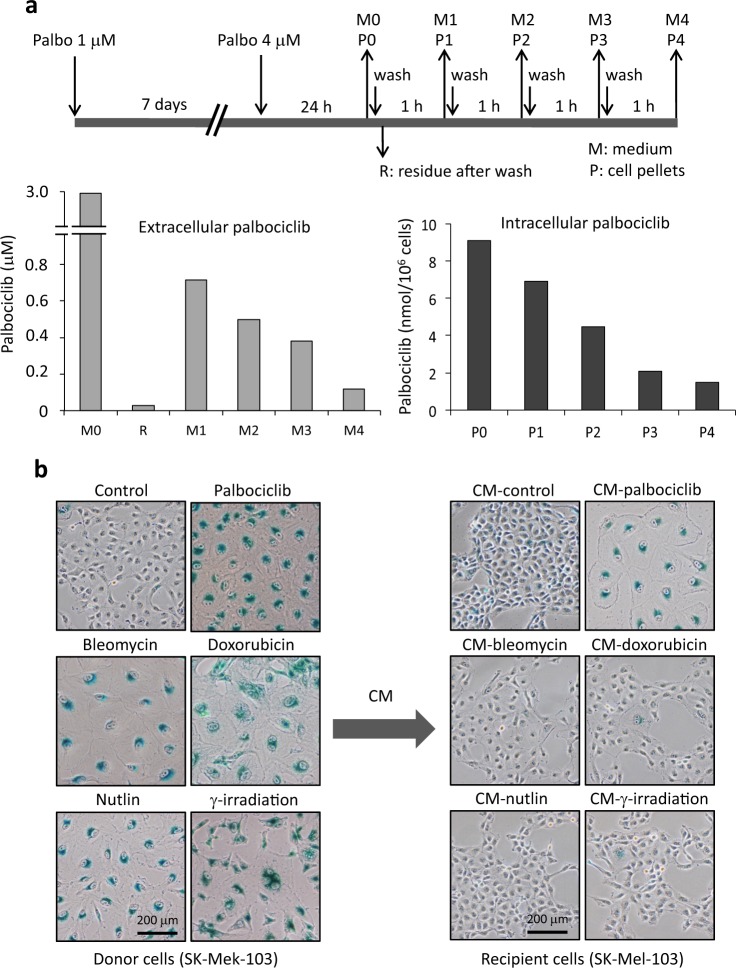
Paracrine senescence by release of lysosome-trapped palbociclib. **a** Senescent SK-Mel-103 cells (exposed to palbociclib 1 µM for 7 days) where counted and seeded in 10 cm dishes (600,000 cells per dish) in the presence of 4 µM palbociclib. 24 h later cells washed with PBS and added fresh media lacking palbociclib. This procedure was repeated every hour for 4 consecutive hours. Cell pellets (P) and conditioned media (M) were collected as illustrated in the diagram and the palbociclib content was assessed by mass spectrometry. Control pellets (P0) and media (M0) were collected prior to the first wash and residual media (R) was collected immediately after washing the dishes for the first time. The values correspond to a representative experiment from a total of two independent experiments. **b** Donor SK-Mel-103 cells were treated with the indicated senescence-inducing stimuli, namely, palbociclib (1 µM), bleomycin (12 mUnits/ml), doxorubicin (10 nM), nutlin (10 µM), and γ-irradiation (10 Gy). The different drugs were added only once and the culture media were not changed for the length of the treatment (7 days). To obtain conditioned media, the senescent cells were washed with PBS and incubated with fresh media for 3 additional days. The media were then collected, filtrated with 0.45 μM filters, diluted 1:2 with fresh media, and added to the recipient cells. Recipient SK-Mel-103 were treated with the conditioned media of donor cells for 7 days, with no media changes, and then stained for SA-βgal activity

We wondered if, similarly to other lysosomotropic drugs, palbociclib can diffuse from the lysosomes out to the extracellular medium. For this, we washed senescent SK-Mel-103 cells with PBS in a sequential manner from 1 to 4 times, at 1 h intervals (Fig. 4a), and we quantified the intracellular and extracellular concentrations of the drug by mass spectrometry. In this experiment, senescent cells were pre-treated with 4 µM palbociclib 24 h before measurements to maximize the intracellular concentration of the drug and facilitate its detection (the exact concentration measured by mass spec was 3.81 µM). The residual (R) palbociclib in drug-free media freshly added immediately after washing was 28 nM, which is below the minimal concentration (125–150 nM) required to induce senescence in SK-Mel-103 cells (Figure S4a and see below Fig. 6b and S6a, d). Importantly, one hour later, the concentration of palbociclib in the medium (M1) had increased to 700 nM (25 times). This concentration was progressively diminished after subsequent washes at 1 h intervals (M2, M3, and M4) (Fig. 4a). We also measured the amount of palbociclib trapped within the cells (P1, P2, P3, and P4). Consistent with our above observations showing that intracellular palbociclib fluorescence progressively disappears when cells are flowed with drug-free media (Fig. 1d), the intracellular concentration of palbociclib also decreased progressively with subsequent changes of media (Fig. 4a). Finally, we also tested if palbociclib can be trapped by the plastic of the culture plates and released upon washing, however we could not detect any palbociclib by mass spectrometry in the wash out media of cell-free plates that had been exposed to the drug (data not shown). Together, these observations demonstrate that palbociclib-treated cells release the drug to the extracellular medium.

To determine if the palbociclib released to the extracellular milieu could have a biological impact, we examined the senescence-inducing capacity of the conditioned media (CM) obtained from senescent SK-Mel-103 cells. Specifically, senescent cells induced with different stimuli, including palbociclib 1 µM, were washed with PBS, incubated with fresh media for 3 additional days, and the resulting media (diluted 1:2 with fresh medium) was added to recipient SK-Mel-103 cells. Interestingly, only palbociclib-CM induced a robust proliferation arrest and senescence in the recipient cells, whereas none of the other conditioned media (from bleomycin, doxorubicin, nutlin, or γ-irradiated senescent cells) efficiently induced senescence in this setting (Fig. 4b and S4b). We reproduced these results with human primary fibroblast (IMR90 cells) (Figure S4c and S4d) thus demonstrating that these effects are not restricted to cancer cells.

### Palbociclib-mediated paracrine senescence is independent of senescence induction

**Fig. 5:**
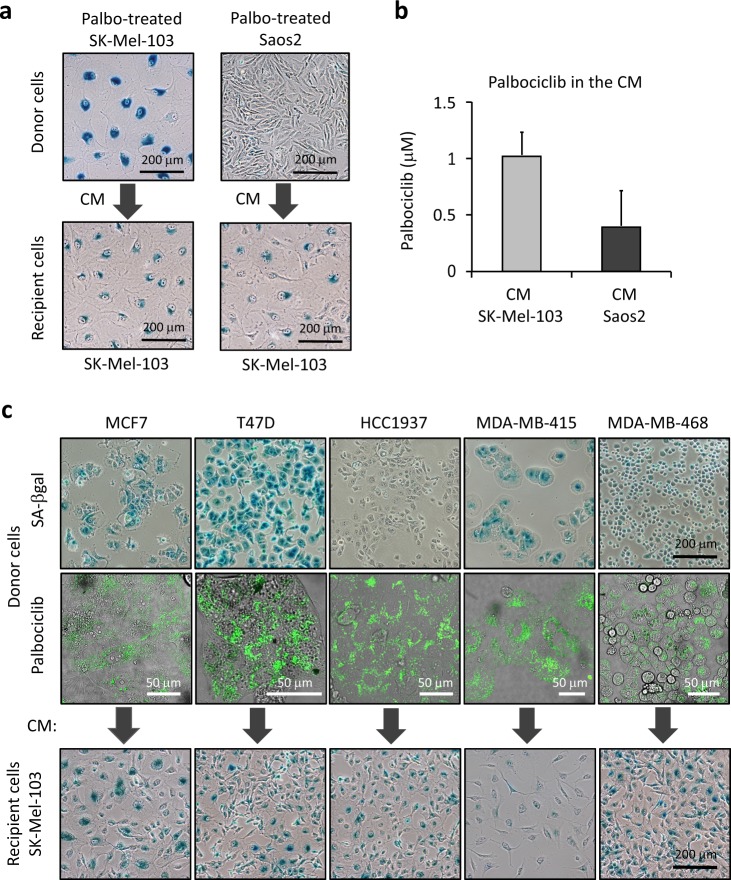
Paracrine senescence can be mediated by palbociclib non-responsive donor cells. **a** Donor SK-Mel-103 and Saos2 cells were treated with palbociclib (4 µM), for 7 days. The drug was added only once and the culture media were not changed for the length of the treatment. To obtain conditioned media, SK-Mel-103 and Saos2 cells were tripsinized and identical numbers of both cell lines were seeded in new dishes in the presence of palbociclib. 24 h later the cells were washed with PBS and incubated with fresh media for 3 additional days. The media were then collected, filtrated with 0.45 μM filters, diluted 1:2 with fresh media, and added to the recipient cells. Recipient SK-Mel-103 were treated with the conditioned media for 7 days, with no media changes, and then stained for SA-βgal activity. **b** Mass spectrometry quantification of palbociclib concentration in the conditioned media of the indicated donor cells treated as in **a**. The graph represents the average values and SD of three independent experiments. **c** Donor palbociclib responsive (MCF7, T47D, and MDA-MB-415) and non-responsive (HCC1937 and MDA-MB-415) human breast cancer cell lines were treated with palbociclib 1 μM for 7 days. The drug was added only once and the culture media were not changed for the length of the treatment. Following the treatment, the cells were tripsinized and seeded in new dishes to be stained for SA-βgal activity (top images) and analyzed by confocal imaging to evaluate the presence of palbociclib (middle images). Prior to image acquisition cells were incubated with 4 µM palbociclib 1 h to improve palbociclib detection. In parallel, conditioned media from identical number of palbociclib-treated cells were obtained as in **a** and added to recipient SK-Mel-103 for 7 days, with no media changes, prior to assessing SA-βgal activity (bottom images)

We have shown above that palbociclib also accumulates in cells that are resistant to palbociclib-induced senescence, such as Saos2 cells (Figure S1h). Based on this, we hypothesized that palbociclib-resistant cells should also produce paracrine senescence through the release of palbociclib to the extracellular medium. Saos2 and SK-Mel-103 cells were treated with palbociblib 4 µM and incubated for 7 days; after this, cells were washed with PBS and incubated with drug-free media for 3 additional days. The resulting conditioned media (diluted 1:2 with fresh medium) was added to recipient SK-Mel-103 cells for 7 days. Interestingly, the palbociclib-CM from Saos2 cells induced paracrine senescence on recipient SK-Mel-103 cells (Fig. 5a). Analysis by mass spectrometry of the palbociclib-CM from SK-Mel-103 and Saos2 cells revealed the presence of palbociclib in the conditioned medium of both cell lines (Fig. 5b). The concentration of palbociclib in the CM of Saos2 cells was lower than in the CM of SK-Mel-103. This difference is likely due to the fact that Saos2 cells do not experience the expansion of the lysosomal compartment that is characteristic of senescent cells and, in fact, accumulate less palbociclib compared to SK-Mel-103 cells (Figure S1h). Still, the concentration of palbociclib detected in the conditioned media of Saos2 cells (~0.5 µM) was sufficient to induce senescence in recipient SK-Mel-103 cells (even if diluted 1:2) (Figure S4a). Taken together, the above observations demonstrate that cells resistant to palbociclib-induced arrest and senescence, can release palbociclib to the extracellular medium in sufficient amounts to produce paracrine senescence in responsive cells.

Palbociclib has been approved for the treatment of metastatic breast cancer. For this reason, we considered of relevance to extend our findings to cell lines derived from these type of tumors. With this aim, we exposed both palbociclib responsive (MCF7, T47D, and MDA-MB-415) [32] and palbociclib resistant (HCC1937 and MDA-MB-468) [32, 33] human breast cancer cell lines to 1 μM palbociclib for 7 days (Fig. 5c, top images). In all cases, we could detect intracellular accumulation of palbociclib by confocal imaging (Fig. 5c, middle images). Moreover, and in agreement with our previous observations, the conditioned media from both responsive and non-responsive cell lines induced senescence in recipient SK-Mel-103 cells (Fig. 5c, bottom images). Next, we extended our observations to palbociclib-responsive human cancer cell lines from different origins (lung: H226; colon: Huh7; head and neck: SCC42B). As expected, the conditioned medium from these cell lines was able to induce paracrine proliferation arrest (Figure S5a) and senescence (Figure S5b) on a variety of palbociblib-responsive cells. These results prove that lysosomal trapping of palbociclib and its paracrine release are general cellular properties independent of the execution or not of senescence.

### Chloroquine potentiates the activity of palbociclib by reducing its lysosomal entrapment

**Fig. 6:**
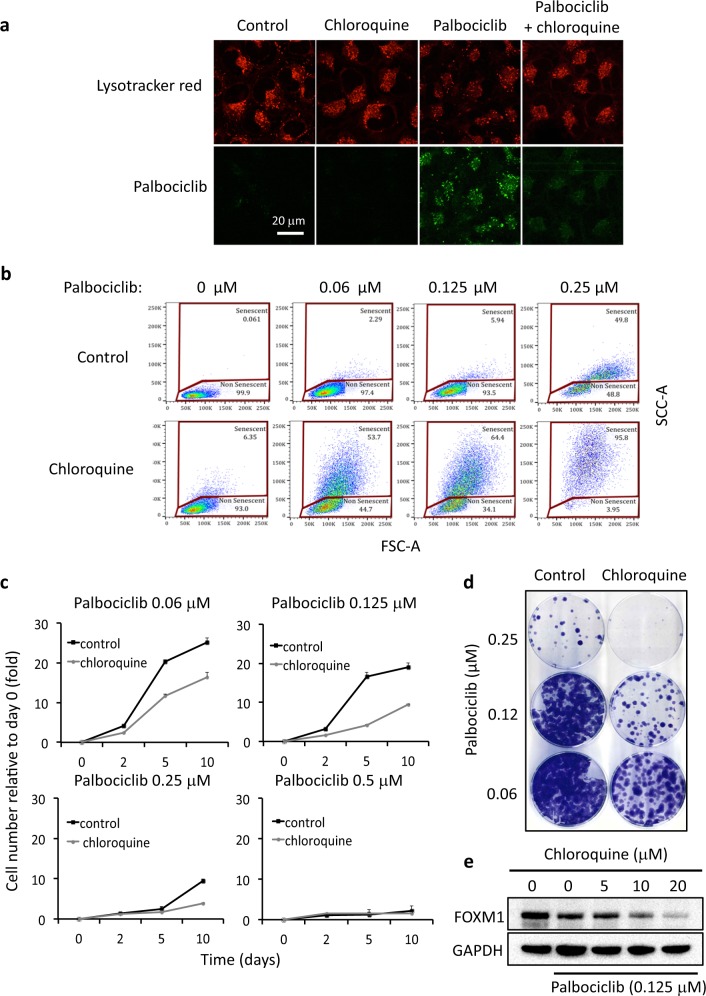
Chloroquine potentiates palbociclib effects by reducing its lysosomal entrapment. **a** Confocal images of the lysotracker red and palbociclib fluorescence of SK-Mel-103 cells treated with 2 μM palbociclib and/or 50 µM chloroquine for 1 h. **b** Flow cytometry analysis (FCS-A vs SSC-A) of the percentage of senescent SK-Mel-103 cells incubated with the indicated concentrations of palbociclib in the presence or absence of 5 μM chloroquine for 7 days. **c**, **d** SK-Mel-103 cells were incubated for 7 days with the indicated concentrations of palbociclib in the presence or absence of 5 μM chloroquine. **c** Following the treatment, identical number of cells were re-seeded in 96-well plates and viable cells were measured at different days to analyze their proliferative capability. Values correspond to a single experiment with four biological replicas. Each condition was normalized to its respective signal at day 0. **d** In addition, 1000 cells from each treatment were re-seeded in 35 cm dishes and 10 days later colony growth was assessed by crystal violet staining. **e** SK-Mel-103 cells were co-incubated for one hour with 0.125 μM palbociclib and the indicated concentrations of chloroquine. The protein levels of the CDK4 substrate FOXM1 were subsequently analyzed by Western blot

When a cell is exposed simultaneously to two different lysosomotropic compounds they compete for intra-lysosomal protonation and their relative lysosomal accumulation depends on their relative concentrations and their respective pKa values [22]. We wondered if the presence of another lysosomotropic compound, such as chloroquine, would reduce the accumulation of palbociclib in the lysosome. To address this, SK-Mel-103 cells were exposed simultaneously to palbociclib and chloroquine. Interestingly, the presence of chloroquine caused a noticeable reduction in the lysosomal accumulation of palbociclib (Fig. 6a). Since the biological action of palbociclib on CDK4/6 occurs in the cytosolic and nuclear compartments, we speculated that its displacement from lysosomes could have a positive impact on its activity. For the following assays, we used a concentration of 5 µM chloroquine, which is within the range of serum concentrations achieved in clinical applications of chloroquine as immunosuppressant [34]. Cells treated simultaneously with chloroquine plus low concentrations of palbociclib underwent senescence more efficiently that those treated with palbociclib alone (Fig. 6b and S6a). Also, cell proliferation and clonal growth were more efficiently reduced by palbociclib when treatments were performed in the presence of chloroquine (Fig. 6c, d). These evidences suggest that chloroquine cooperates with palbociclib in the induction of cell-cycle arrest and senescence. Of note, however, cells treated with chloroquine and palbociclib did not display a prominent SA-βgal activity (Figure S6a) and, therefore, the cellular outcome may be better described as an atypical senescence. Chloroquine reduces the acidic pH of lysosomes (see for example Fig. 1a) and this may reduce the activity of lysosomal β-galactosidase. Finally, we measured the effect of chloroquine on the ability of palbociclib to inhibit CDK4/6. For this, we used a suboptimal amount of palbociclib that only partially reduced the levels of FOXM1 and, under these conditions, addition of chloroquine further reduced FOXM1 levels (Fig. 6e).

To further strengthen the concept of synergism between palbociclib and lysosomtropic agents, we tested the effect of ammonium chloride (NH_4_Cl), another lysosomotropic compound that increases the internal pH value of lysosomes (Figure S6b). We observed that, similarly to chloroquine, the presence of NH_4_Cl (pKa = 9.24) prevented the lysosomal accumulation of palbociclib (Figure S6c) and promoted cell size enlargement similar to that of senescent cells, although, as it happened before with chloroquine, cells were negative or weakly positive for SA-βgal activity (Figure S6d). Finally, we quantified by mass spectrometry the intracellular concentration of palbociclib, which mostly corresponds to the lysosomal pool, and found that it was noticeably reduced when palbociclib was co-incubated with either chloroquine or NH_4_Cl (Figure S6e). Therefore, lysosomotropic compounds, such as chloroquine and NH_4_Cl, can reduce the lysosomal entrapment of palbociclib, thus increasing its availability in the cytoplasmic and nuclear compartments where it exerts its inhibitory actions on CDK4/6.

### Lysosomal entrapment of the CDK4 inhibitors abemaciclib and ribociclib

Ribociclib and abemaciclib are two other clinically approved CDK4/6 inhibitors. Like palbociclib, these compounds contain protonatable amine groups and pKa values above 8 (Figure S7a). These similar chemical properties led us to speculate that abemaciclib and ribociclib could also have lysosomotropic properties. We first confirmed that the two compounds induced senescence in SK-Mel-103 cells, although their relative potencies varied, being abemaciclib more potent than palbociclib, and ribociclib being the less potent (Figure S7b). We also observed that ribociclib was fluorescent when excited with emission light of 405 nm, although its emission profile differed slightly from palbociclib (Figure S7c). Ribociclib-fluorescence was also observed by microscopy (collecting light with a filter of 450–500 nm) (Figure S7d) and its intracellular pattern was indistinguishable from that of lysosomes (visualized with lysotracker) (Figure S7e). We could not observe any fluorescent signal in cells exposed to abemaciclib with the experimental settings applied for the detection of either palbociclib (filter of 500–550 nm) or ribociclib (filter of 450–500 nm) (Figure S7d).

**Fig. 7:**
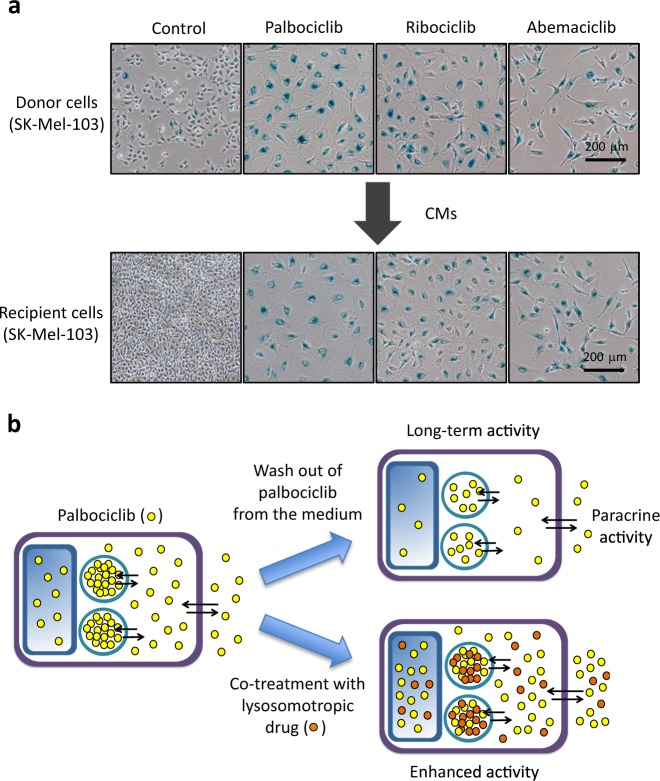
Lysosomal trapping is a feature common to all CDK4 inhibitors. **a** Donor SK-Mel-103 cells were treated with 1 μM palbociclib, 1 μM abemaciclib or 1 μM ribociclib for 7 days. The drugs were added only once and the culture media were not changed for the length of the treatment. Following treatments, cells were tripsinized and identical numbers were seeded in new dishes in the presence of the drugs. 24 h later the dishes were washed with PBS and incubated with fresh media for 3 additional days. The media were then collected, filtrated with 0.45 μM filters, diluted 1:2 with fresh media, and added to the recipient cells. Recipient SK-Mel-103 were incubated with the conditioned media for 7 days, with no media changes, and then stained for SA-βgal activity. **b** The reversible storage of palbociclib into lysosomes (left) has three main implications: (top right) it prolongs the availability of the drug (long-term activity) and induces paracrine effects (paracrine activity); (bottom right) the presence of another lysosomotropic drug, reduces the accumulation of palbociclib into lysosomes and this increases its cytoplasmic concentration thereby potentiating its intracellular activity (enhanced activity) as well as its extracellular release (paracrine activity)

To test whether abemaciclib or ribociclib are lysosomotropic, we conditioned media from senescent SK-Mel-103 cells exposed to these drugs (1 μM) for 7 days, washed with PBS, and incubated with fresh media for 3 additional days. Supporting our hypothesis, all conditioned media had the capability to induce senescence (Fig. 7a). These observations strongly suggest that lysosomal trapping is a property common to all three clinically approved CDK4/6 inhibitors.

## Discussion

Cellular acidic organelles play a key role in various cellular processes such as the turnover of phospholipids, the breakdown of endogenous waste products, autophagy, and apoptosis. Weakly basic and lipophilic drugs often accumulate into lysosomes through a non-enzymatic ion trapping mechanism known as lysosomal trapping. In this work, we show that palbociclib is reversibly trapped into lysosomes and, based on this, we demonstrate novel experimental properties of palbociclib that may be relevant to understand its pharmacology (Fig. 6b). We found that other CDK4 inhibitors: ribociclib and abemaciclib share with palbociclib the chemical properties that cause their lysosomal trapping and therefore our findings can be potentially extended to these compounds. In addition, we report the intrinsic fluorescence properties of palbociclib, which greatly facilitates its detection in lysosomal stores. This property is shared with ribociclib, which is structurally related to palbociclib, but not with abemaciclib.

It has been reported that certain drugs can be retained by the plastic surface of tissue culture dishes and released after washing out the medium [35]. However, by mass spectrometry, we could not detect any palbociclib in the wash out media of cell-free plates that had been exposed to the drug (data not shown).

The establishment of senescence is known to require a sustained activation of the senescence-inducing pathways [30, 31]. In this regard, the prolonged release of lysosomal-trapped palbociclib to the cytosolic and nuclear compartments may be critical in producing a sustained inhibition of CDK4/6. In addition, lysosomal-trapped palbociclib is not only released to the cytosol but also extracellularly, where it may act in a paracrine manner. This is particularly relevant when non-responsive cells trap and release palbociclib, as we have shown here. In a clinical setting, lysosomal trapping of palbociclib may significantly contribute to the engagement of senescence in responsive cells due to the long-term release of the drug from both responsive and non-responsive cells. We also show that ribociclib and abemaciclib are released into the extracellular medium of drug-treated cells, being able to produce paracrine senescence in a manner similar to palbociclib. Another potential beneficial aspect of lysosomal trapping is that it may reduce drug toxicities on normal tissues [36].

A negative aspect of the lysosomal sequestration of palbociclib is the reduction of the cytosolic and nuclear concentration achieved by the drug within cells, which may be responsible for the increased cellular and therapeutic IC50 with regard to the biochemical value. Indeed, lysosomal trapping has been described as a mechanism of resistance for some drugs [37]. In vivo, lysosomal trapping also reduces the systemic availability of amphiphilic drugs and significantly contributes to the total uptake of drugs by tissues [38–40]. This is particularly relevant in the case of orally-administered drugs that are trapped by the liver, a phenomenon known as pre-systemic metabolism [41–43]. Weakly basic drugs can alter the capacity of lysosomes to accumulate co-administered lysosomotropic drugs [22, 44]. In agreement with this, we have found that chloroquine, a weak basic anti-malarial and immunosuppressant drug, and NH_4_Cl reduce the accumulation of palbociclib in lysosomes and potentiate its cellular effects. The diminished accumulation of palbociclib into lysosomes translates into an increased bioavailability in its target subcellular compartments, (nucleus and cytosol), and a reduction in the minimal concentration of palbociclib required to induce cell cycle arrest and senescence. In vivo, lysosomotropic compounds, such as chloroquine and NH_4_Cl, may also displace a drug of interest from lysosomal-rich organs, such as lungs, liver, and kidneys, to the tumor [38–40]. Therefore, the combination of palbociclib with lysosomotropic drugs could improve its therapeutic efficacy. Indeed, it has been reported that chloroquine and palbociclib synergize in reducing solid tumor growth in nude mice, an effect that was ascribed to the inhibition of autophagy by chloroquine [45]. In addition to this, and based on our current data, chloroquine may also promote the release of palbociclib from the lysosomal stores resulting in a more efficient inhibition of CDK4/6.

In summary, we show that palbociclib undergoes lysosomal trapping and this has antagonistic consequences: it increases the exposure time to the drug, but it reduces its peak concentration in the cytosol and nucleus (Fig. 7b). In addition, the lysosomal trapping of palbociclib mediates the paracrine effects of both responding and non-responding cells (Fig. 7b). The above-described effects are likely to impact on the pharmacokinetics of palbociclib, including its effective concentration, its volume of distribution, and its half-life. Finally, we present suggestive evidence indicating that these concepts also apply to the two other clinically approved CDK4/6 inhibitors, namely, ribociclib and abemaciclib.

## Materials and methods

### Fluorescence spectroscopy analysis of palbociclib

Palbociclib was weighed and dissolved in milli-Q water at the indicated concentrations. The pH of the dilutions was adjusted to either pH 4.5 or pH 7.5 with HCl. Each condition was assayed in triplicate in 96-well dark plates using a Synergy Hi microplate reader (BioTek). Fluorescence spectra was obtained using an excitation wavelength of 405 nm. Gen5TM software was used for data collection, and Prism 7 software was used for data plotting.

### Senescence induction

For senescence induction SK-Mel-103 cells were treated with 1–4 μM palbociclib (Selleckchem), 1 μM ribociclib (Selleckchem), 1 μM abemaciclib (Absource Diagnostics GmbH), 10 nM doxorubicin (Sigma), bleomycin 12 mUnits/ml (Selleckchem), 10 μM nutlin-3 (Sigma), or γ-irradiated (10 Gy). Drugs were added only once and the culture media were not changed for the length of the treatment.

### Senescence-associated β-galactosidase (SA-βgal) activity

Senescence-associated β-galactosidase (SA-βgal) activity was assessed with the senescence β-galactosidase staining kit from Cell Signaling (cat # 9860) following the manufacturer’s instructions.

### Confocal microscopy

Confocal images were acquired in a TCS-SP5 AOBS laser scanning confocal microscope (Leica Microsystems). For the detection of palbociclib fluorescence in live cells we used a 405 nm excitation laser and collected the emitted light between 500 and 550 nm. For the detection of ribociclib fluorescence in live cells we used a 405 nm excitation laser and collected the emitted light between 450 and 500 nm. Acridine orange and lysotracker red (both from Life Technologies) were added at 1 μg/ml to the culture media 30 min before image acquisition. To acquire the lysotracker signal live cells were excited with a 561 nm laser and the fluorescence was detected in the 580–650 nm range. Acridine Orange signal was acquired by excitation of live cells with an Argon laser (488 nm) and the emitted signal was collected in two separate fluorescence emission ranges: from 500 to 550 and from 660 to 750 nm. To analyze the effect of lysosomotropic compounds on the pH of acidic vesicles, 4 μM palbociclib (Sellekchem), 50 μM chloroquine (Sigma) or 40 nM bafilomycin (Calbiochem) were added to the cells 1 h before acridine orange addition. The fluorescence signal of palbociclib and lysotracker red in individual cells was quantified using a Defineas software. At least 100 cells of each conditions were quantified.

### Flow cytometry

For the detection of palbociclib fluorescence profile, we used a Gallios Flow Cytometer (Beckman Coulter) and an excitation laser of 405 nm. For cell cycle profiling cells were seeded at sub-confluency in 60 mm plates. Following the indicated treatment, floating and attached cells were collected and fixed in 70% ethanol. Fixed cells were treated with RNAse 100 μg/ml (Qiagen) and stained with propidium iodide (SigmaAldrich) and DAPI (SigmaAldrich). Subsequently the cell cycle distribution was evaluated in a FACSscalibur cytometer (BD Biosciences, Franklin Lakes, NJ). For the quantification of cellular senescence, cells were trypsinized and resuspended in PBS buffer. Live control and senescent cells were differentiated on the basis of their scatter pattern (FCS-A vs SSC-A) using a FACS CANTO II (BD, San Jose, CA) for the assessment of the percentage of senescent cells and a FACS ARIA IIu for cell sorting experiments. At least 10,000 live events were collected for each condition. The analysis of all flow cytometry data was performed using FlowJo v10 (Treestar, OR) software applying an identical gating strategy for all the experiments: we used pulse processing to exclude cell aggregates and DAPI (Sigma) to exclude dead cells. To discriminate control and senescent cells we plotted FSC vs SSC and used untreated and senescent cells (treated with 1 μM palbociclib for 7 days) as reference for each independent experiment.

### Conditioned media

Following the senescence-inducing treatment, cells were washed with PBS and incubated with fresh media. 3 days later the media were collected and filtrated with 0.45 μM filters, diluted 1:2 with fresh media, and added to the recipient cells. When the conditioned media were obtained from different cell types or when the different treatments resulted in noticeably dissimilar number of cells (Figs. 5a, c and 7a), 24 h before conditioning the media, the cultures were tripsinized and identical number of cells were seeded in the presence of the corresponding drugs.

### Serial washes

Following incubation with palbociclib (24 h) the cells were washed with PBS and fresh drug-free media was added to the tissue culture plates. This procedure was repeated for the indicated number of times with intervals of either 30 (Fig. 3a) or 60 (Fig. 4a) minutes between subsequent changes of media.

### Microfluidics experiment

Palbociclib release from lysosomes was tested both on SK-Mel-103 and Saos2 cells. The cells were pre-treated with 1 µM palbociclib for 7 days and then seeded in a 6-channels flow chamber slides (IBIDI μSlide IV) in the presence of 4 μM palbociclib. The following day, a flux assay (200 μl/min) was performed simultaneously in two channels, injecting in one channel drug-free media and palbociclib (4 µM) in the other one. For the analysis of palbociclib uptake SK-Mel-103 were pre-treated with 1 µM palbociclib for 7 days and then seeded in a 6-channels flow chamber slides (IBIDI μSlide IV) in the absence of palbociclib. The following day, media containing palbociclib (4 µM) was continuously fluxed through the cultures (200 μl/min). Pictures were taken every 10 minutes with a DMI6000B microscope (Leica microsystems): 405 nm excitation and detection with a 500–550 nm filter. The fluorescence signal of the cells and the background in each frame was quantified with ImageJ. At least 10 individual cells were analyzed in each experimental condition.

### Cell proliferation assay

Cell number and cell proliferation were analyzed with the CellTiter-Glow Luminescent Cell Viability Assay from Promega following the manufacturer instructions. The cells were seeded in glass bottom multiwell 96 plates (Greiner) and the luminescence was measured on a Victor Multilaber Plate Reader (PerkinElmer).

### Immunoblotting

Cells were harvested in lysis buffer containing 1% SDS and 1% Triton X-100. Identical amounts of protein were resolved by SDS/PAGE and electro-transferred to nitrocellulose membranes. Blots were incubated with the primary antibodies anti-phospho-RB1 Ser 807/811 from Cell Signaling (cat # 9308), anti-FOXM1 (D12D5) from Cell Signaling (cat #5436), anti-β actin anti-p63/SQSTM1 from Novus Biologicals (NBP1–49956) and LAMP1 from Cell Signaling (C54H11), and subsequently incubated with the corresponding HRP-conjugated secondary antibody (Dako). Images were acquired in a ChemiDocMP Imaging System (BioRad).

### Cytokine analysis

The conditioned media of control and senescent cells (72 h) was analyzed with a proteome array containing antibodies against 36 human cytokines from R&D Systems (Proteome profiler human cytokine array kit, ARY005B) following the manufacturer´s instructions.

### Mass spectrometry

LC–MS analysis was conducted using an Agilent 1100 series LC system consisting of a G1312A binary pump, a G1379A degasser and an ALSG1330B refrigerated autosampler interfaced to an API 2000 mass spectrometer. The turboionspray source (TIS) was operated in positive ionization mode. Instrument control and data analysis were performed using Analyst® 1.6.2 application software from Applied Biosystems. The purification of the analytes was performed with a Phenomenex Gemini 3 µm C18 110 Å analytical column with mobile phases consisting on a mixture of acetonitrile with 0.1% formic acid and water with 0.1% formic acid (99:1 v/v). The elution of the analytes was carried out using a flow rate of 0.6 ml/min. Nebulizer and curtain gas pressure were 70 and 20 psi, respectively. LC–MS interface was set at 400 °C; declustering potential (DP), entrance potential (EP) and spray voltages were set at 60, 12, and 5000 V, respectively. At this working voltage, protonated molecular ions were detected with the greatest sensitivity, and these ions were chosen to quantify palbociclib ([M + H] = 448). Mass spectrometric data were collected between 0 and 5 min. For detection of palbociclib, cellular pellets were resuspended in 100 µl of cold acetonitrile/methanol (1:1) and incubated in agitation overnight at 4 °C in order to denature proteins and extract the compound. Conditioned media and cellular samples were processed for solid phase extraction (SPE) on HybridSPE-Phospholipid Small Volume 15 mg/96-Well plate (Supelco, USA) using the following procedures according to the supplier: (1) adding 50 µl of sample to the HybridSPE-PL plate followed by a precipitating agent consisting of 0.1% formic acid in acetonitrile; (2) performing in-well protein precipitation by agitating on an oscillating table and mix for 2 minutes at a setting of 1000 oscillations/minute; (3) transferring the HybridSPE-PL plate to a vacuum manifold, and apply vacuum (−10 to 15 in. Hg); (4) collecting the resulting eluent and 5 µl of the final solution was injected onto the LC column. Calibration curves were prepared in drug-free cell medium with working solutions of palbociclib standards. The range of standard concentrations tested was from 5 to 10,000 ng/ml. The peak area ratios of palbociclib were analyzed by linear regression to estimate the slope, intercept and correlation coefficient of the calibration curve. Standard curves in each analytical run were used to calculate the concentrations of the samples.

### Cells

Cell lines SK-Mel-103 (human melanoma), Saos2 (human osteosarcoma), (HCT116 human colon adenocarcinoma), Huh7 (human hepatocarcinoma), IMR90 (human fetal lung fibroblasts), H226 (lung SCC) were obtained from the American Type Culture Collection and were authenticated after completion of this work. Human breast cancer cell lines: MCF7, T47D, HCC1937, MDA-MB-415, and MDA-MB-468 were provided by Dr. Miguel Angel Quintela. UT-SCC-42B cells (human HNSCC derived from primary lesions) were provided by Dr. Reidar Grenmman and have been previously described [46–49]. Mycoplasma detection tests were carried out periodically in all cell lines.

### Statistical analysis

All the experiments were performed with two to four experimental replicas. When two independent experiments were performed, a representative experiment was shown. The average of the mean of the biological replicates was calculated when three or more independent experiments were carried out. Variation between independent experiments was represented by the standard deviation of the mean. We used the Student’s two-sided *t*-test to examine the statistically significance of the differences. *P* values of ≤ 0.05 were considered to be statistically significant. The number of experimental replicas, the number of experiments, the sample size and the statistical analysis is clearly stated in the corresponding figure legends.

## Supplementary information

Supplementary Figures
