# Supplementary material for: Lysosomal trapping of palbociclib and its functional implications
Source: Oncogene. 2019 Jan 28;38(20):3886–902. doi: 10.1038/s41388-019-0695-8 (PMC6756094; doi:10.1038/s41388-019-0695-8)
Supplement: Supplementary file 1 — Supplementary Figures [file 41388_2019_695_MOESM1_ESM.pdf]

Figure S1

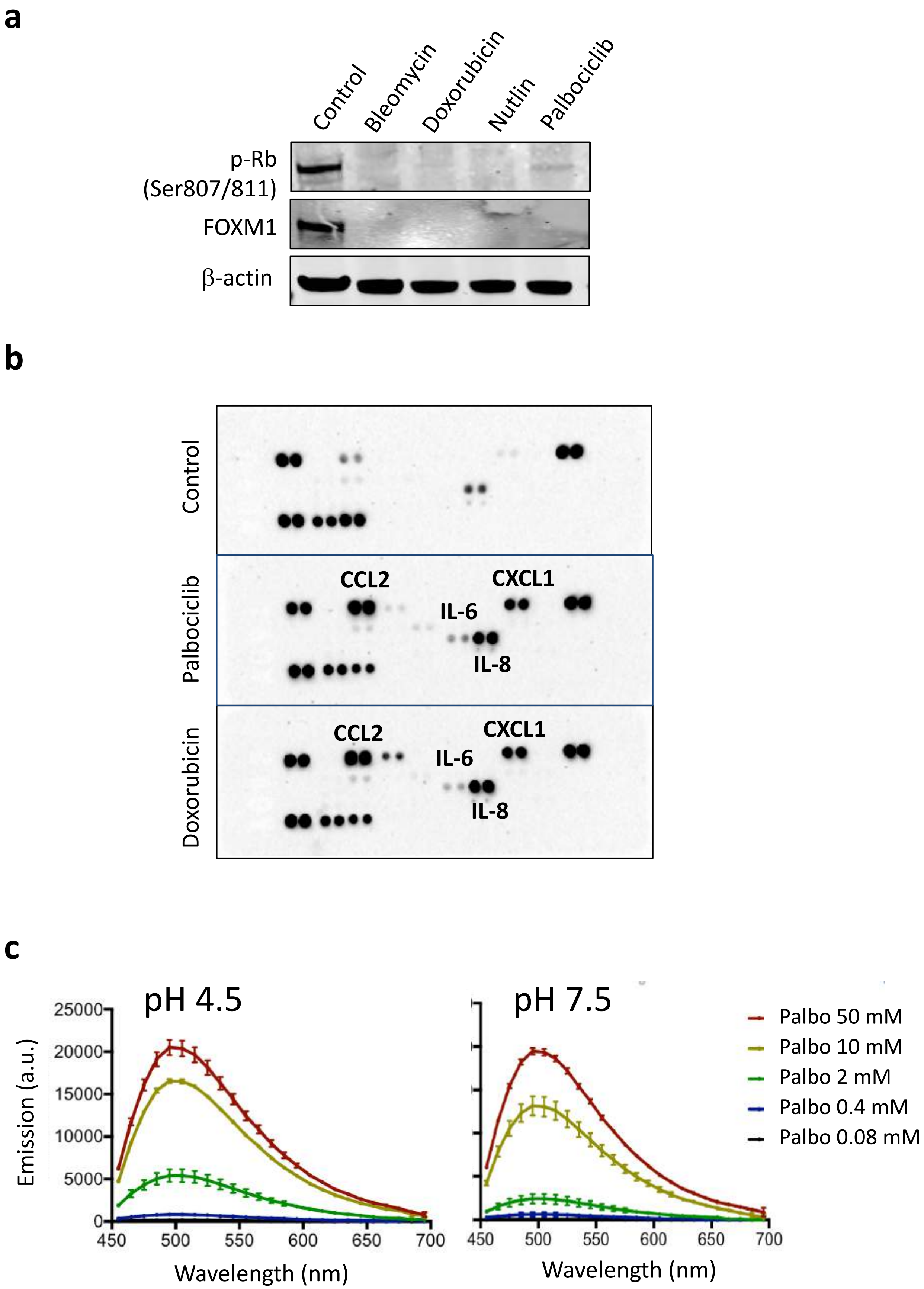
a
Control
Bleomycin
Doxorubicin
Nutlin
Palbociclib
p-Rb
(Ser807/811)
FOXM1
β-actin
b
Control
Palbociclib
Doxorubicin
CCL2
CXCL1
IL-6
IL-8
c
pH 4.5
pH 7.5
Emission (a.u.)
25000
20000
15000
10000
5000
0
450
500
550
600
650
700
Wavelength (nm)
Palbo 50 mM
Palbo 10 mM
Palbo 2 mM
Palbo 0.4 mM
Palbo 0.08 mM

Figure S1

d

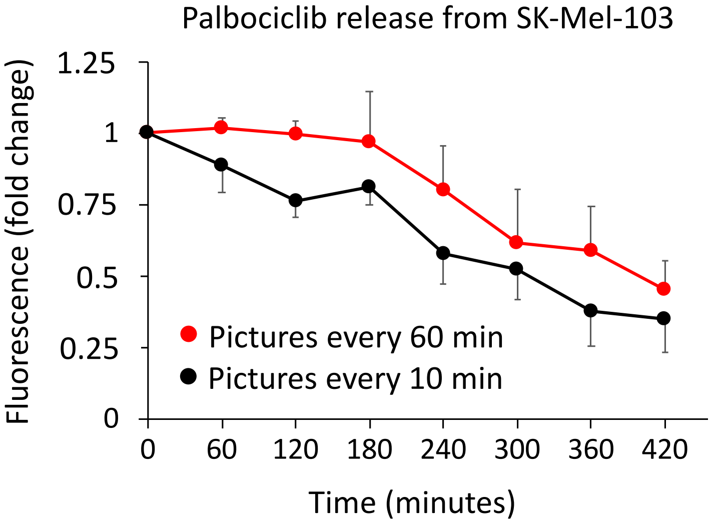

Palbociclib release from SK-Mel-103
Fluorescence (fold change)
1.25
1
0.75
0.5
0.25
0
0
60
120
180
240
300
360
420
Time (minutes)
Pictures every 60 min
Pictures every 10 min

e

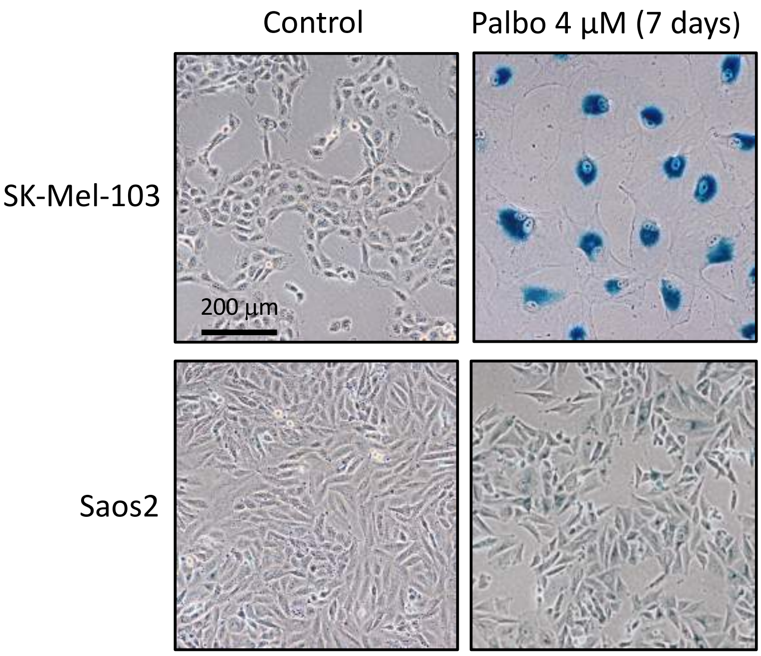

Control
Palbo 4 µM (7 days)
SK-Mel-103
Saos2
200 µm

Figure S1

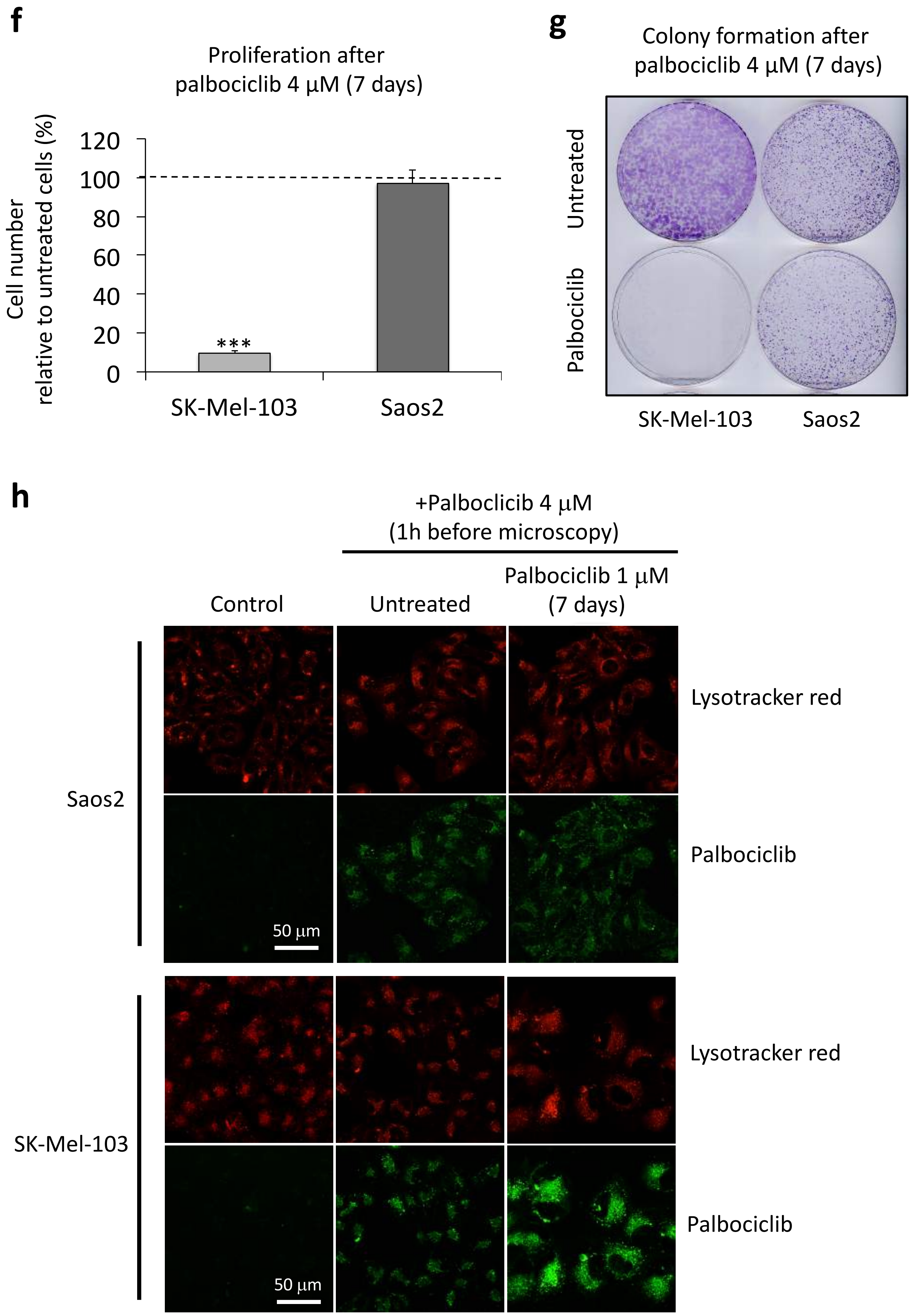
f
Proliferation after
palbociclib 4 μM (7 days)
Cell number
relative to untreated cells (%)
120
100
80
60
40
20
0
***
SK-Mel-103
Saos2
g
Colony formation after
palbociclib 4 μM (7 days)
Untreated
Palbociclib
SK-Mel-103
Saos2
h
+Palbociclib 4 μM
(1h before microscopy)
Control
Untreated
Palbociclib 1 μM
(7 days)
Saos2
SK-Mel-103
Lysotracker red
Palbociclib
Lysotracker red
Palbociclib
50 μm
50 μm

Figure S1

i

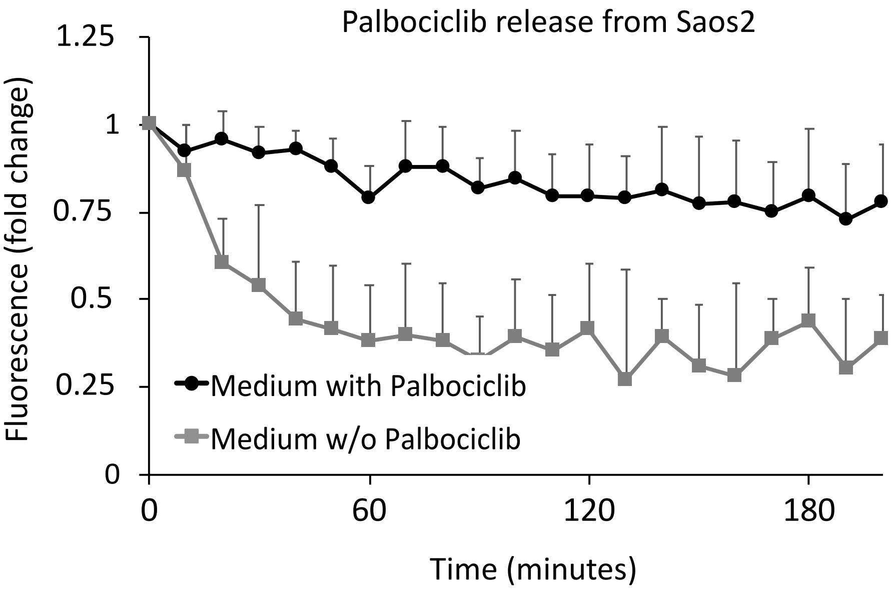

Palbociclib release from Saos2
Fluorescence (fold change)
1.25
1
0.75
0.5
0.25
0
Medium with Palbociclib
Medium w/o Palbociclib
0
60
120
180
Time (minutes)

j

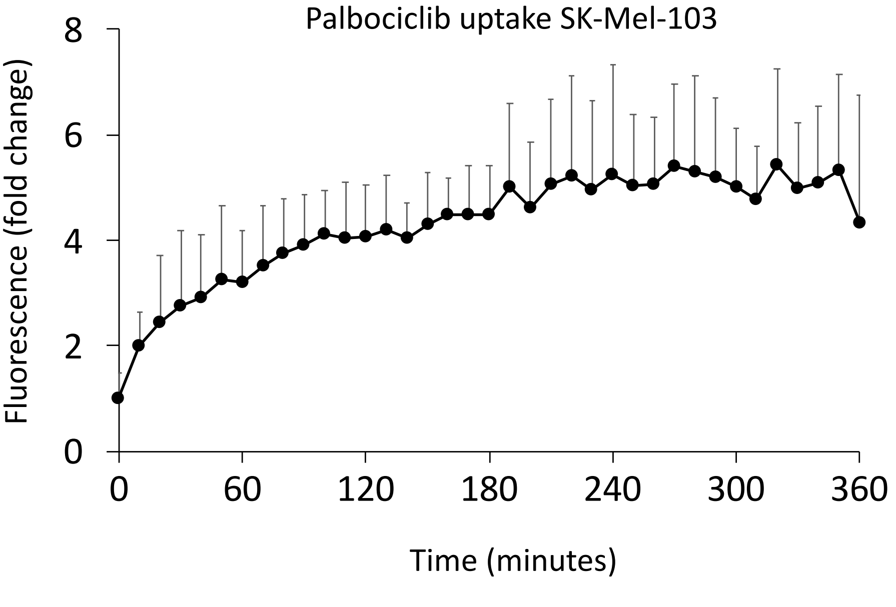

Palbociclib uptake SK-Mel-103
Fluorescence (fold change)
8
6
4
2
0
0
60
120
180
240
300
360
Time (minutes)

Figure S1. (**a**) Western blot analysis of the levels of phosphorylated RB1 (Ser 807/811) and FOXM1 in SK-Mel-103 cells treated with the indicated senescence-inducing drugs (bleomycin 12 mUnits/ml, doxorubicin, nutlin 10 μM, palbociclib 1 μM). The different drugs were added only once and the culture media were not changed for the length of the treatment (7 days). (**b**) Antibody-array detection of cytokines in the conditioned media of SK-Mel-103 control or treated with 1 μM palbociclib or 10 nM doxorubicin for 7 days. (**c**) Emission profile of palbociclib following excitation with a 405 nm laser. The drug was diluted in milli-Q water and the pH was adjusted to either 7.5 or 4.5 with HCl. Values correspond to the average and SD of biological replicates (n=3). (**d**) Palbociclib-fluorescence of live SK-Mel-103 treated with palbociclib 1 μM for 7 days and then seeded in flow chamber slides in the presence 4 μM palbociclib. 24 hours after seeding, chamber slides were flowed with palbociclib-free medium and pictures were taken either every 10 minutes (the complete experiment is shown in Figure 1d) or every 60 minutes. Values represent the average and SD of palbociclib-fluorescence in 10 individual cells corrected by the background and normalized to time 0. (**e**) SA-βgal activity of SK-Mel-103 and Saos2 cells untreated or treated with palbociclib 4 μM, for 7 days. (**f**) Saos2 and SK-Mel-103 cells control or treated with palbociclib 4 μM, for 7 days, were re-seeded in 96 well plates in the absence of drug, allow to proliferate for 4 days, and cell viability was measured. Values correspond to the average and SD of biological replicates (n=3) normalized to untreated cells. Statistical *t*-test analysis was performed to calculate significance (***$P \leq 0.005$). (**g**) Saos2 and SK-Mel-103 cells untreated or treated with palbociclib 4 μM, for 7 days, were re-seeded in 35 cm dishes (1,000 cells per dish) in the absence of drug. Colonies were visualized by crystal violet staining 10 days after seeding. (**h**) Confocal images of Saos2 and SK-Mel-103 cells either untreated or treated with palbociclib 4 μM, for 7 days. Cells were incubated in the absence (control) or presence of palbociclib 4 μM for 1 hour prior to image acquisition. (**i**) Analysis of palbociclib-fluorescence in live Saos2 cells treated with palbociclib 1 μM for 7 days and then seeded in flow chamber slides in the presence 4 μM palbociclib. 24 hours after seeding, chamber slides were flowed with either palbociclib-free medium or palbociclib-containing medium (4 μM) and pictures were taken either every 10 minutes. (**j**) Palbociclib-fluorescence in live SK-Mel-103 treated with palbociclib 1 μM for 7 days and then seeded in flow chamber

slides in the absence of palbociclib. 24 hours after seeding, chamber slides were flowed with medium containing palbociclib 4 μM and pictures were taken either every 10 minutes. In (i) and (j) values represent the average and SD of palbociclib-fluorescence in 10 individual cells corrected by the background and normalized to time 0.

Figure S2

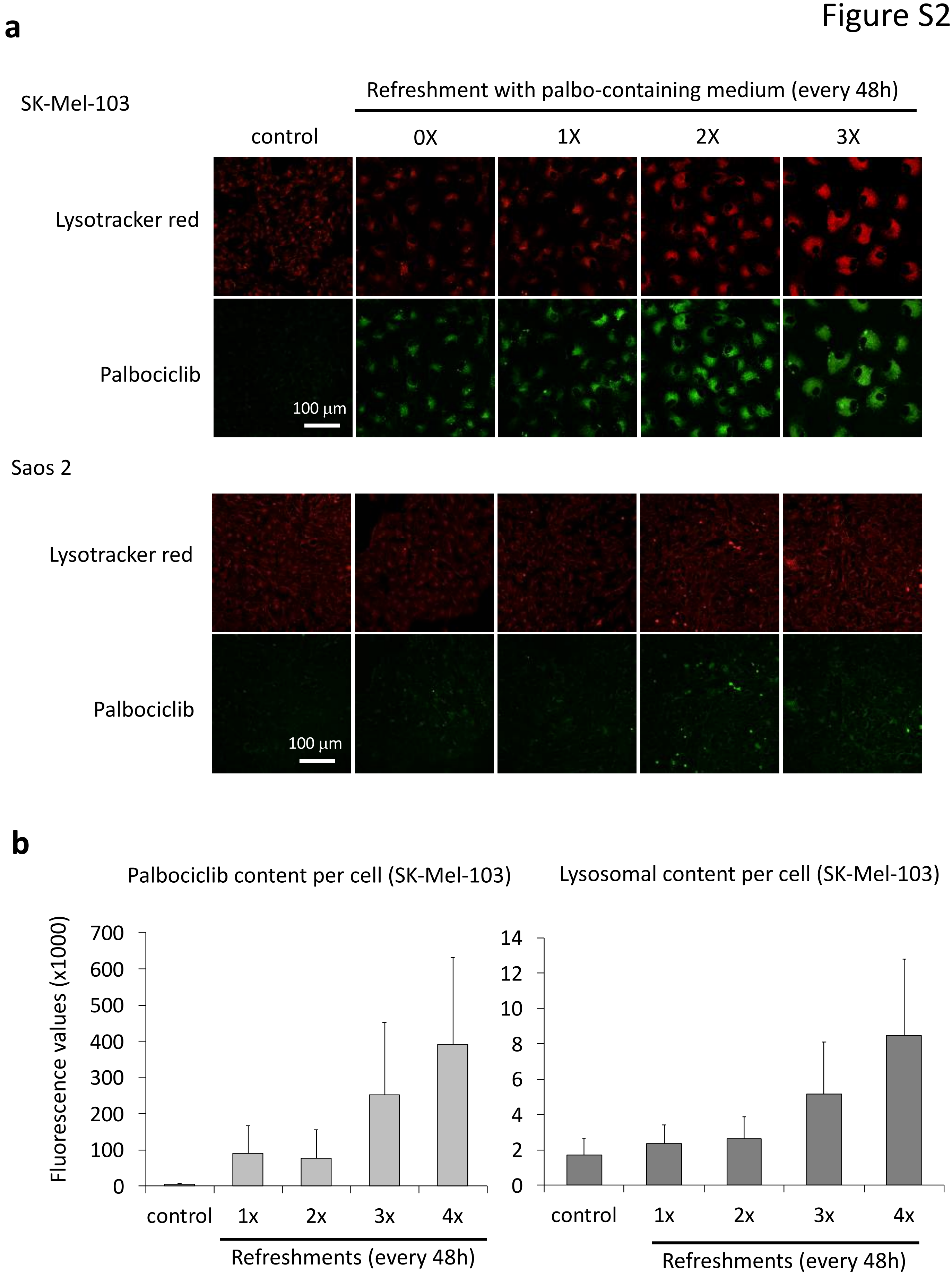

a
SK-Mel-103
Refreshment with palbo-containing medium (every 48h)
control
0X
1X
2X
3X
Lysotracker red
Palbociclib
100 µm
Saos 2
Lysotracker red
Palbociclib
100 µm
b
Palbociclib content per cell (SK-Mel-103)
Lysosomal content per cell (SK-Mel-103)
Fluorescence values (x1000)
700
600
500
400
300
200
100
0
14
12
10
8
6
4
2
0
control
1x
2x
3x
4x
Refreshments (every 48h)
control
1x
2x
3x
4x
Refreshments (every 48h)

Figure S2. (**a**) Representative confocal images of SK-Mel-103 and Saos2 cells treated with 1 μM palbociclib for 7 days. In 1x, the culture medium was substituted by fresh medium containing 1 μM palbociclib after 48 h and then left until day 7. In 2x, the culture medium was replaced after 48h and then, again, after the subsequent 48 h. In 3x, there were a total of 3 changes every 48 h. The images were acquired 7 days after the first addition of palbociclib. (**b**) Quantification of the fluorescence signal of palbociclib and lysotracker red in the confocal images of SK-Mel-103 cells acquired in the experiment shown in (a).

Figure S3

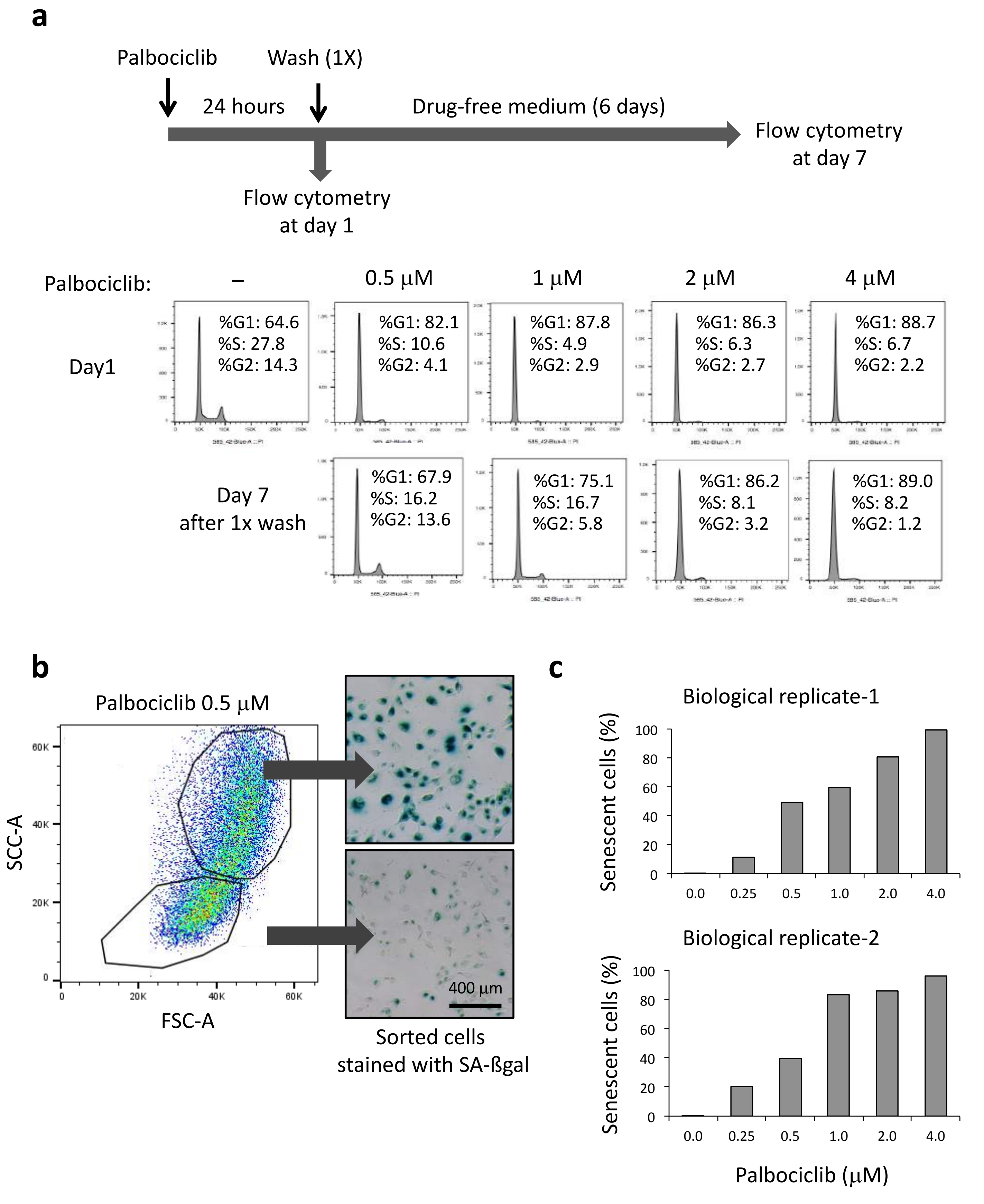
a
Palbociclib
Wash (1X)
24 hours
Drug-free medium (6 days)
Flow cytometry at day 7
Flow cytometry at day 1
Palbociclib:
–
0.5 µM
1 µM
2 µM
4 µM
Day1
%G1: 64.6 %S: 27.8 %G2: 14.3
%G1: 82.1 %S: 10.6 %G2: 4.1
%G1: 87.8 %S: 4.9 %G2: 2.9
%G1: 86.3 %S: 6.3 %G2: 2.7
%G1: 88.7 %S: 6.7 %G2: 2.2
Day 7 after 1x wash
%G1: 67.9 %S: 16.2 %G2: 13.6
%G1: 75.1 %S: 16.7 %G2: 5.8
%G1: 86.2 %S: 8.1 %G2: 3.2
%G1: 89.0 %S: 8.2 %G2: 1.2
b
Palbociclib 0.5 µM
SCC-A
FSC-A
0
20K
40K
60K
400 µm
Sorted cells stained with SA-ßgal
c
Biological replicate-1
Biological replicate-2
Senescent cells (%)
0
20
40
60
80
100
0.0
0.25
0.5
1.0
2.0
4.0
Palbociclib (µM)

d

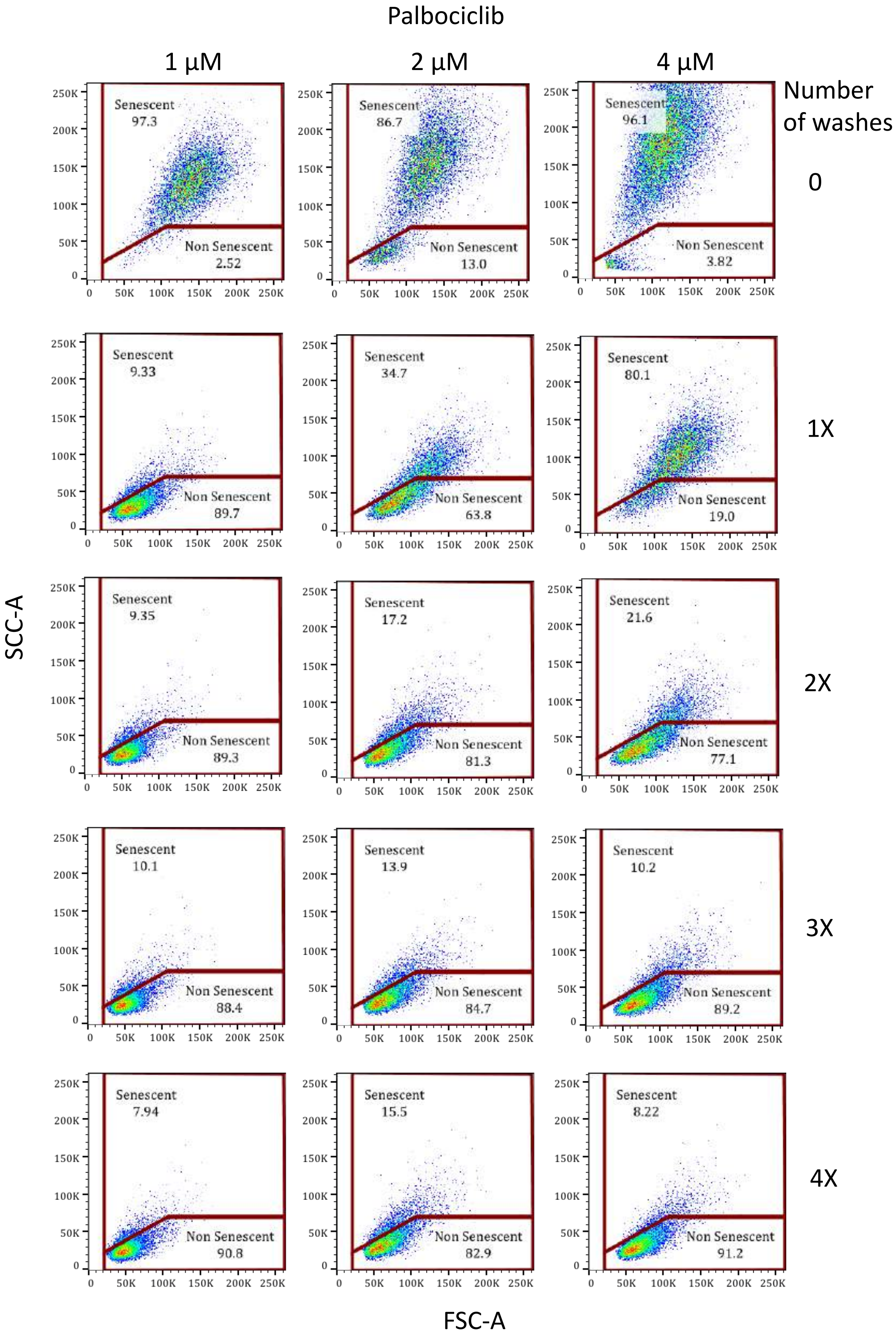
Palbociclib
1 µM
2 µM
4 µM
Number of washes
0
1X
2X
3X
4X
SCC-A
FSC-A
Senescent 97.3
Non Senescent 2.52
Senescent 86.7
Non Senescent 13.0
Senescent 96.1
Non Senescent 3.82
Senescent 9.33
Non Senescent 89.7
Senescent 34.7
Non Senescent 63.8
Senescent 80.1
Non Senescent 19.0
Senescent 9.35
Non Senescent 89.3
Senescent 17.2
Non Senescent 81.3
Senescent 21.6
Non Senescent 77.1
Senescent 10.1
Non Senescent 88.4
Senescent 13.9
Non Senescent 84.7
Senescent 10.2
Non Senescent 89.2
Senescent 7.94
Non Senescent 90.8
Senescent 15.5
Non Senescent 82.9
Senescent 8.22
Non Senescent 91.2

Figure S3

e

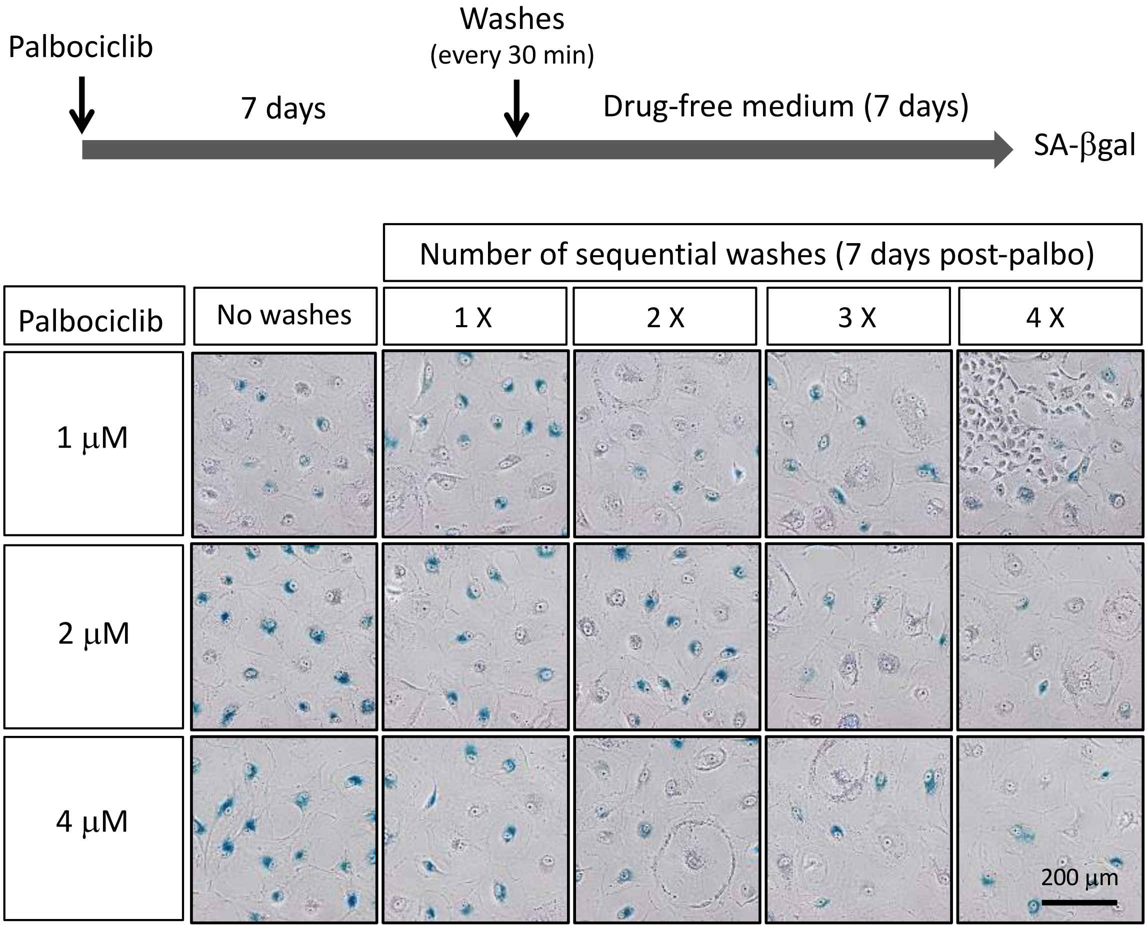

Palbociclib
Washes
(every 30 min)
7 days
Drug-free medium (7 days)
SA-βgal
Number of sequential washes (7 days post-palbo)
Palbociclib
No washes
1 X
2 X
3 X
4 X
1 μM
2 μM
4 μM
200 μm

Figure S3. (**a**) Flow cytometry analysis of SK-Mel-103 cells treated with the indicated concentrations of palbociclib for 24 hours (top row of cell cycle profiles) and then washed with PBS and incubated in the absence of palbocicib for 6 additional days (bottom row of cell cycle profiles). (**b**) SA-βgal activity of control and senescent SK-Mel-103 cells sorted by flow cytometry. Cells were treated with 0.5 μM palbociclib for 7 days. The drug was added once and the culture media was not changed for the duration of the treatment. Subsequently, cells were sorted by their scatter values (FCS-A vs SSC-A) using as reference untreated cells and cells exposed to 1 μM palbociclib for 7 days. The SA-βgal activity was assessed 24 h after sorting. (**c**) Quantification of senescent cells in SK-Mel-103 cultures exposed to increasing concentrations of palbociclib for 7 days and analyzed by flow cytometer measurement of their scatter patterns (FCS-A vs SSC-A). The data from two biological replicates are shown. (**d**) Representative experiment depicting the quantification by flow cytometry (FCS-A vs SSC-A) of senescent SK-Mel-103 cells treated as in Figure 3a. SK-Mel-103 cells were exposed to different concentrations of palbociclib for 24 h. Following palbociclib treatment, cells were washed with PBS and they were added fresh media lacking palbociclib. This procedure was repeated for the indicated number of times. Between successive changes of media, cultures were resting for 30 minutes. Flow cytometry analysis was performed 7 days after palbociclib withdrawal. A total of two biological replicates were performed: one is shown here and the other one is shown (only the numerical data) in Figure 3b. (**e**) SK-Mel-103 cells were treated with different concentrations of palbociclib for 7 days. The drug was added only once and the culture medium was not changed for the duration of the treatment. Ensuing palbociclib treatment, the cells were washed with PBS and were added fresh media lacking palbociclib. This procedure was repeated for the indicated number of times with 30 minutes incubation intervals between consecutive changes of media. The SA-βgal activity was assessed 7 days after palbociclib withdrawal.

Figure S4

a

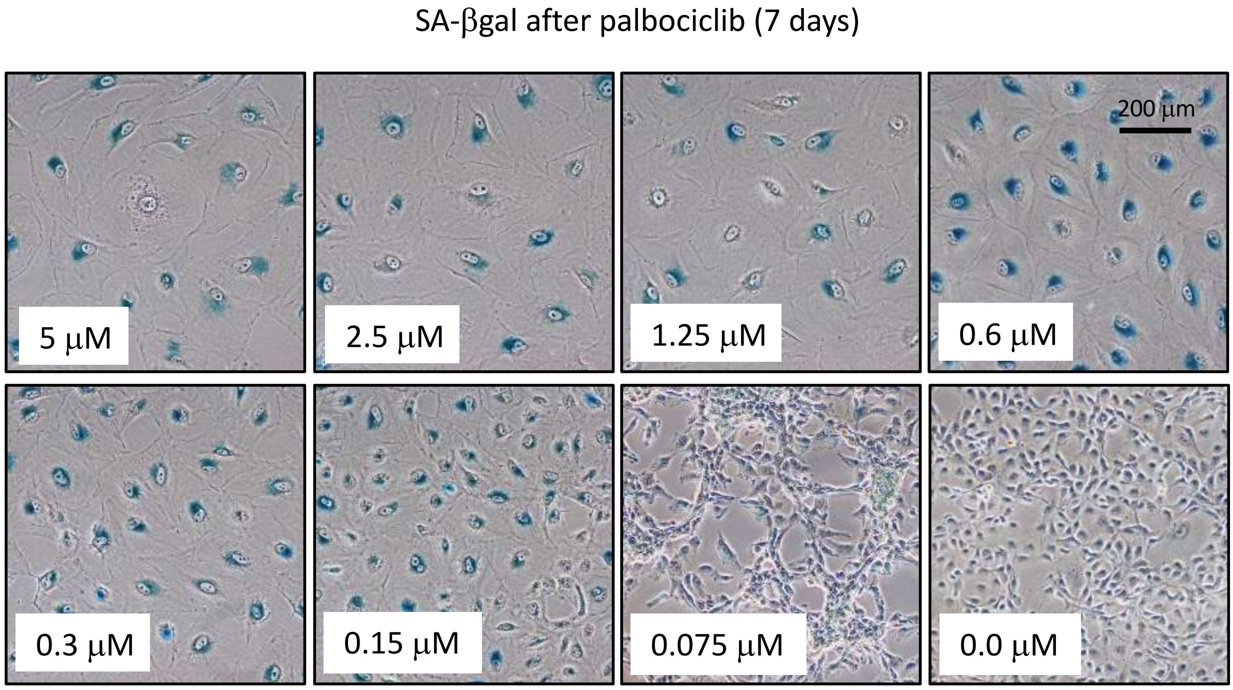
SA-βgal after palbociclib (7 days)
200 μm
5 μM
2.5 μM
1.25 μM
0.6 μM
0.3 μM
0.15 μM
0.075 μM
0.0 μM

b

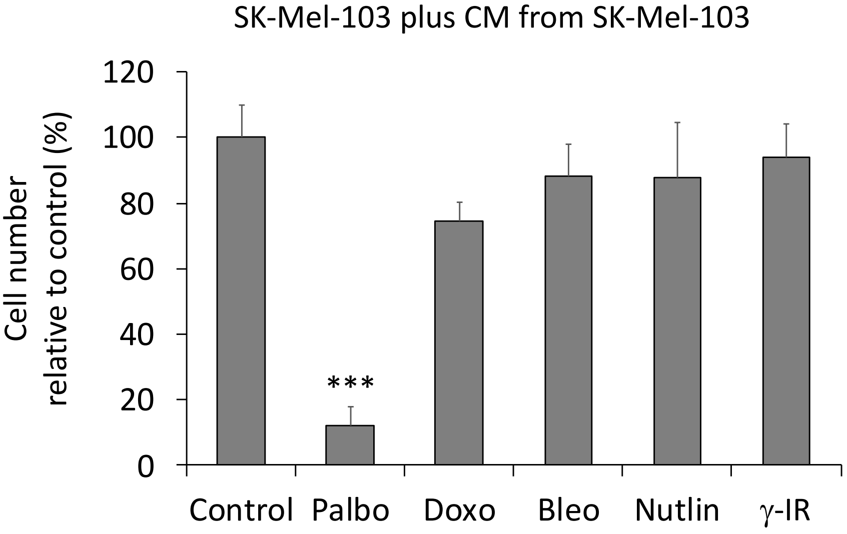
SK-Mel-103 plus CM from SK-Mel-103
Cell number relative to control (%)
120
100
80
60
40
20
0
***
Control
Palbo
Doxo
Bleo
Nutlin
γ-IR

Figure S4

c

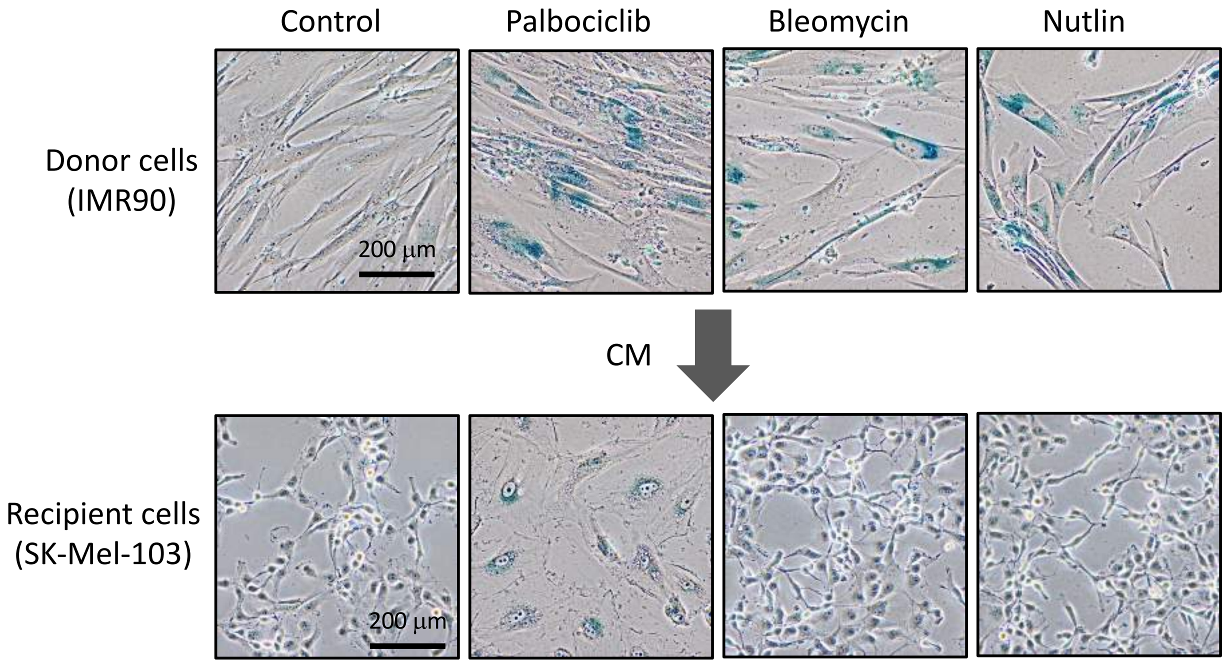
Control
Palbociclib
Bleomycin
Nutlin
Donor cells
(IMR90)
200 μm
CM
Recipient cells
(SK-Mel-103)
200 μm

d

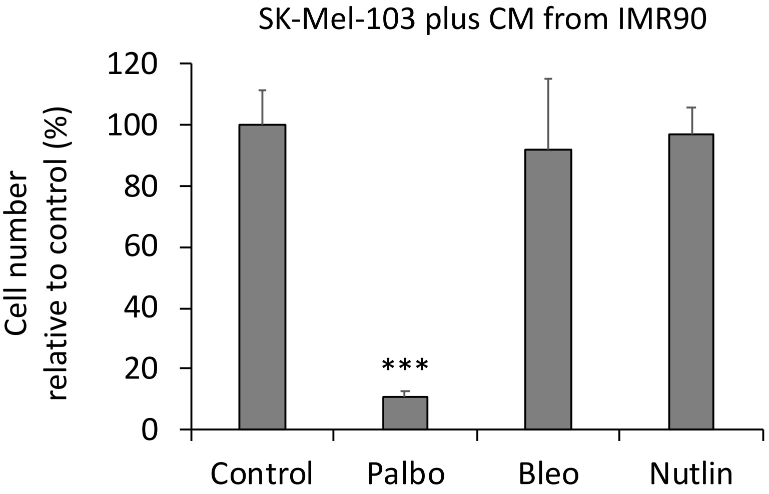
SK-Mel-103 plus CM from IMR90
Cell number
relative to control (%)
120
100
80
60
40
20
0
***
Control
Palbo
Bleo
Nutlin

Figure S4. (**a**) SA-βgal activity of SK-Mel-103 cells following incubation with the indicated concentrations of palbociclib for 7 days. (**b**) Quantification of the cell proliferation of recipient SK-Mel-103 cells ensuing 4 days incubation with conditioned media from either control or senescent SK-Mel-103 cells. To induce senescence SK-Mel-103 cells were treated with the indicated senescence-inducing stimuli for 7 days. Namely, palbociclib (2 μM), bleomycin (12 mUnits/ml), doxorubicin (10 nM), nutlin (10 μM), and γ-irradiation (10 Gy). The different drugs were added only once and the culture media were not changed for the length of the treatment. The values correspond to the average and SD of three independent experiments normalized to untreated control cells. Statistical *t*-test analysis was performed to calculate significance (***$P \leq 0.005$) relative to control. (**c**) SA-βgal activity of donor IMR90 cells (top images) and recipient SK-Mel-103 cells (bottom images). Donor cells were treated with the indicated senescence-inducing stimuli for 7 days. Namely, palbociclib (2 μM), bleomycin (12 mUnits/ml) and nutlin (10 μM). The different drugs were added only once and the culture media were not changed for the length of the treatment. To obtain conditioned media, the senescent cells were washed with PBS and incubated with fresh media for 3 additional days. The media were then collected, filtrated with 0.45 μm filters, diluted 1:2 with fresh media, and added to the recipient cells. Recipient cells were incubated with the conditioned media of donor cells for 7 days, without media changes, before assessing their SA-βgal activity. (**d**) Quantification of the cell proliferation of recipient SK-Mel-103 cells ensuing 4 days incubation with conditioned media from either control or senescent IMR90 cells. Values correspond to the average and SD of three independent experiments normalized to untreated cells. Statistical *t*-test analysis was performed to calculate significance (***$P \leq 0.005$).

Figure S5

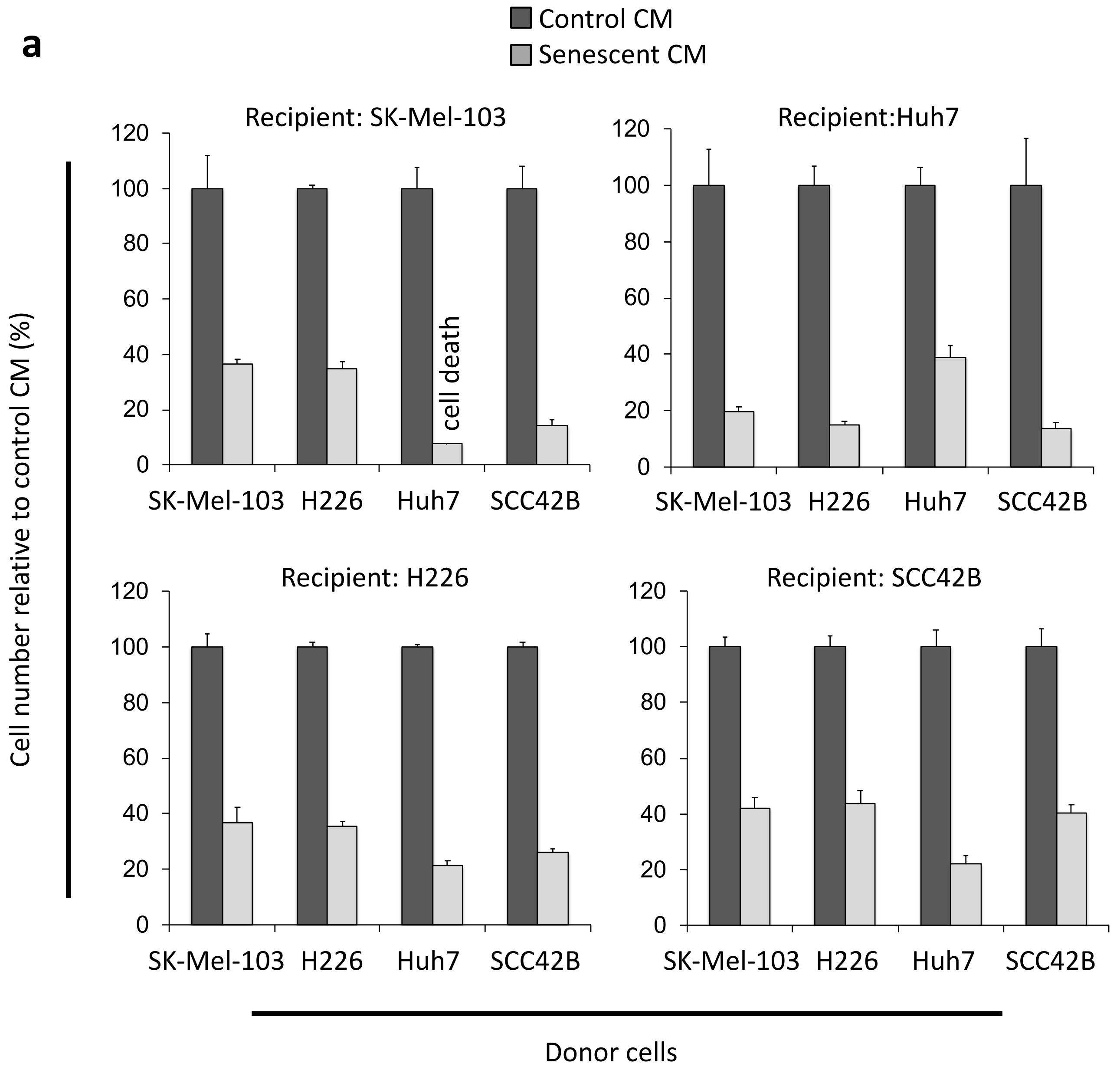
a
Control CM
Senescent CM
Recipient: SK-Mel-103
Recipient:Huh7
Recipient: H226
Recipient: SCC42B
cell death
Cell number relative to control CM (%)
0
20
40
60
80
100
120
SK-Mel-103
H226
Huh7
SCC42B
Donor cells

Figure S5

b

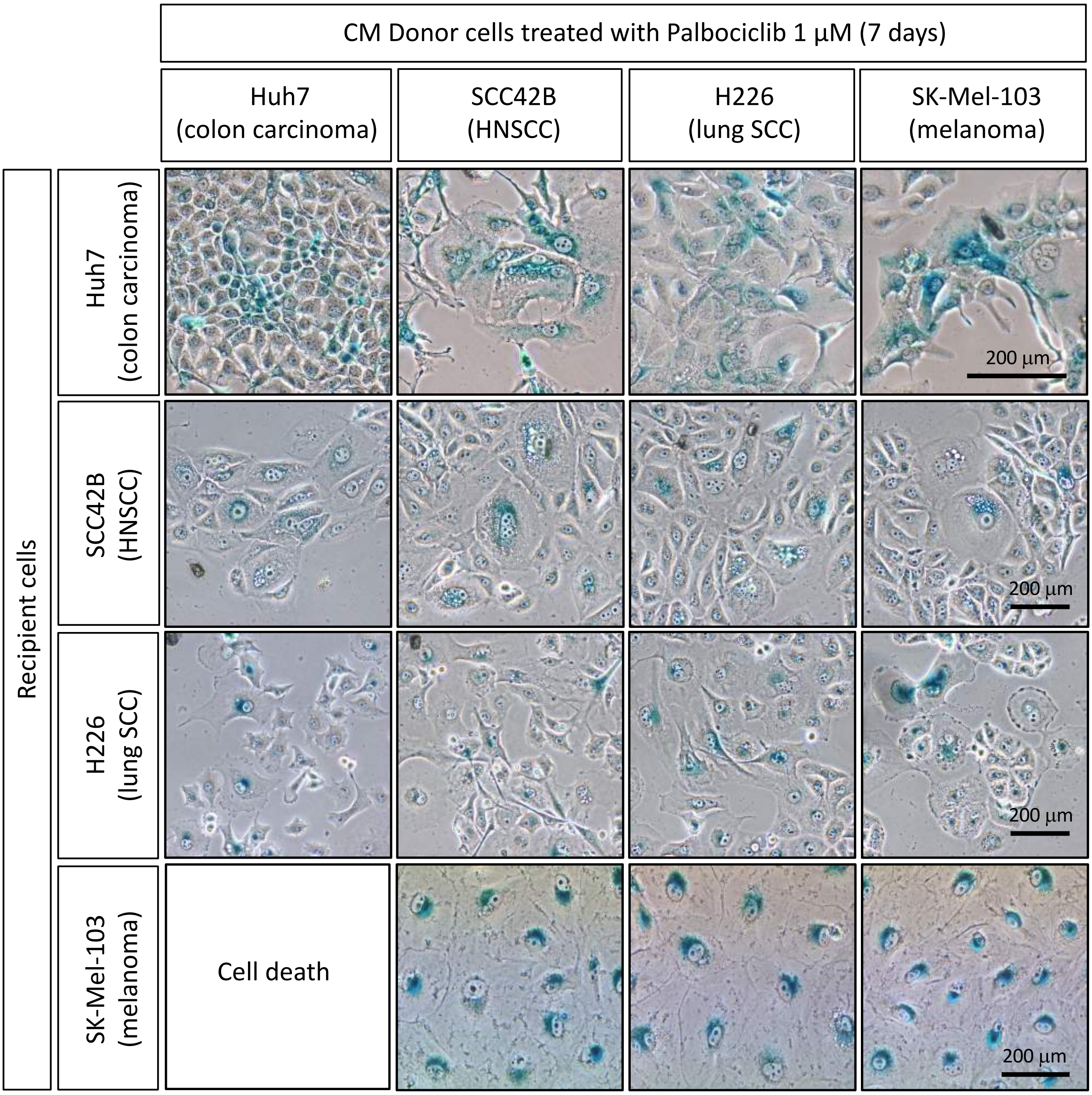

CM Donor cells treated with Palbociclib 1 μM (7 days)
Huh7 (colon carcinoma)
SCC42B (HNSCC)
H226 (lung SCC)
SK-Mel-103 (melanoma)
Recipient cells
Huh7 (colon carcinoma)
SCC42B (HNSCC)
H226 (lung SCC)
SK-Mel-103 (melanoma)
Cell death
200 μm
200 μm
200 μm
200 μm

Figure S5. (**a**) Quantification of the cell proliferation of recipient SK-Mel-103, H226, Huh7 and SCC42B cells ensuing 4 days incubation with conditioned media from either control or senescent donor SK-Mel-103, H226, Huh7 and SCC42B cells. Donor cells were incubated with 2 μM palbociclib for 7 days. Palbociclib was added only once and the culture media were not changed for the length of the treatment. Data correspond to a representative experiment. (**b**) SA-βgal activity of donor and recipient SK-Mel-103, H226, Huh7 and SCC42B cells. Donor cells were incubated with 2 μM palbociclib for 7 days. Palbociclib was added only once and the culture media were not changed for the length of the treatment. To obtain conditioned media, the senescent cells were washed with PBS and incubated with fresh media for 3 additional days. The media were then collected, filtrated with 0.45 μm filters, diluted 1:2 with fresh media, and added to the recipient cells. Recipient cells were incubated with the conditioned media of donor cells for 7 days, with not media changes, before assessing their SA-βgal activity.

Figure S6

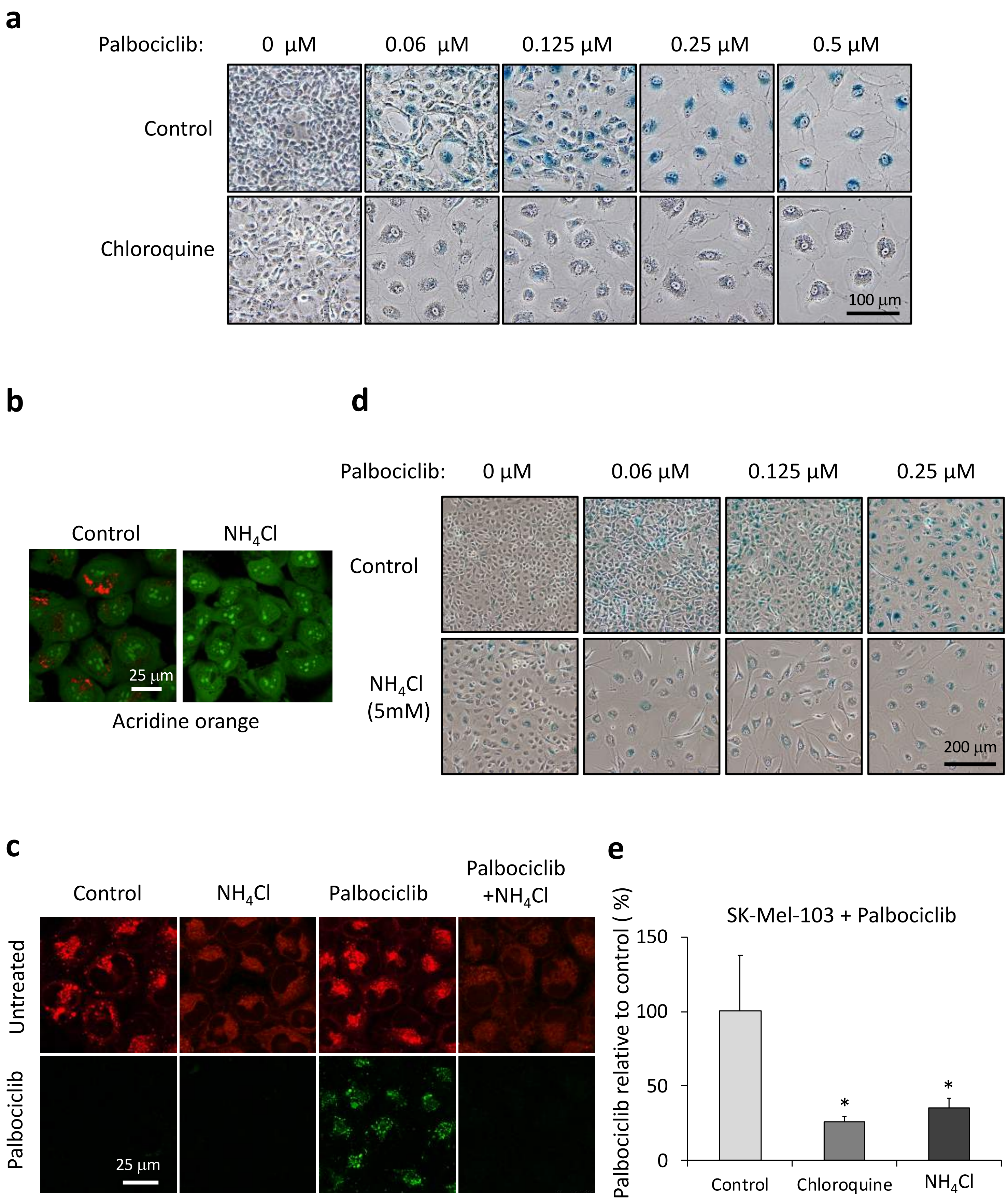
a
Palbociclib:
0 µM
0.06 µM
0.125 µM
0.25 µM
0.5 µM
Control
Chloroquine
100 µm
b
Control
NH_4Cl
25 µm
Acridine orange
d
Palbociclib:
0 µM
0.06 µM
0.125 µM
0.25 µM
Control
NH_4Cl (5mM)
200 µm
c
Control
NH_4Cl
Palbociclib
Palbociclib +NH_4Cl
Untreated
Palbociclib
25 µm
e
SK-Mel-103 + Palbociclib
Palbociclib relative to control (%)
150
100
50
0
Control
Chloroquine
NH_4Cl
*
*

Figure S6. (a) SA-ßgal activity of SK-Mel-103 cells pre-treated with the indicated concentrations of palbociclib in the absence (control) or presence of 5 μM chloroquine for 7 days. Drugs were added only once and the culture media were not changed for the length of the treatment. (**b**) Confocal images of acridine orange-stained SK-Mel-103 cells incubated with acridine orange in the presence or absence of 25 mM NH_4Cl for 1 hour. (**c**) Confocal images of lysotracker red and palbociclib-fluorescence in SK-Mel-103 cells treated with palbociclib (2 μM) in the presence or absence of NH_4Cl (25 mM) for 1 hour. (**d**) SA-βgal activity of SK-Mel-103 cells pre-treated with the indicated concentrations of palbociclib in the absence (control) or presence of 5 mM NH_4Cl for 7 days. Drugs were added only once and the culture media were not changed for the length of the treatment. (**e**) Mass spectrometry quantification of the cellular content of palbociclib in cells exposed for 1 hour to palbociclib (2 μM) in the presence of either chloroquine (5 mM) or NH_4Cl (50 mM). Values correspond to the average and SD of three independent experiments normalized to palbociclib-treated cells. Statistical *t*-test analysis was performed to calculate significance ($*P \leq 0.05$).

Figure S7

a

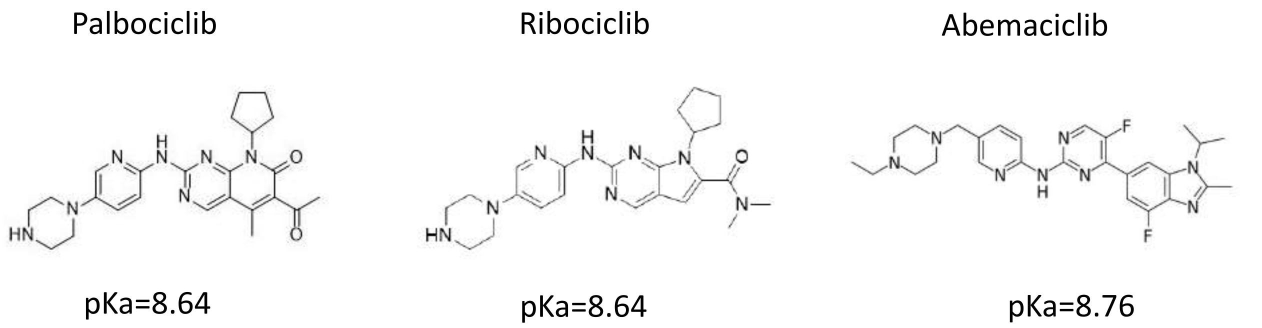

b

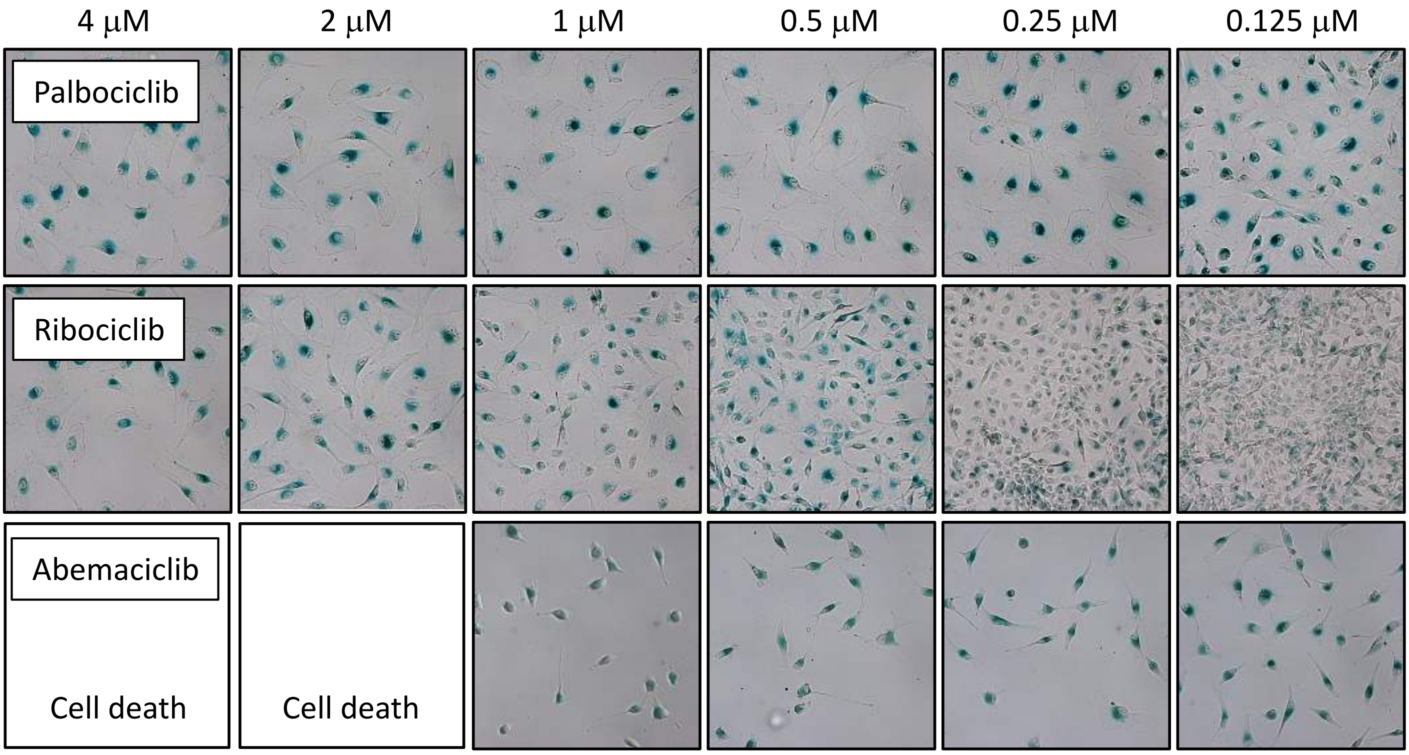

c

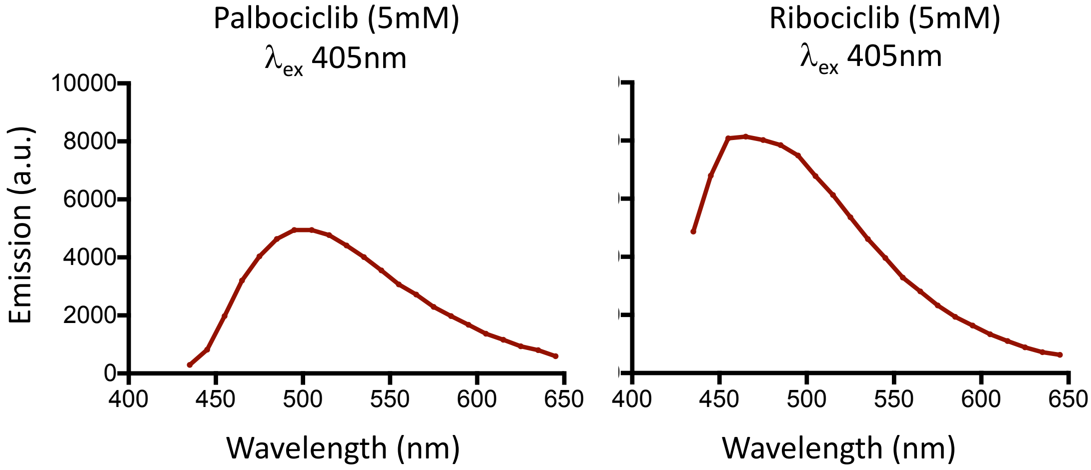

Figure S7

d

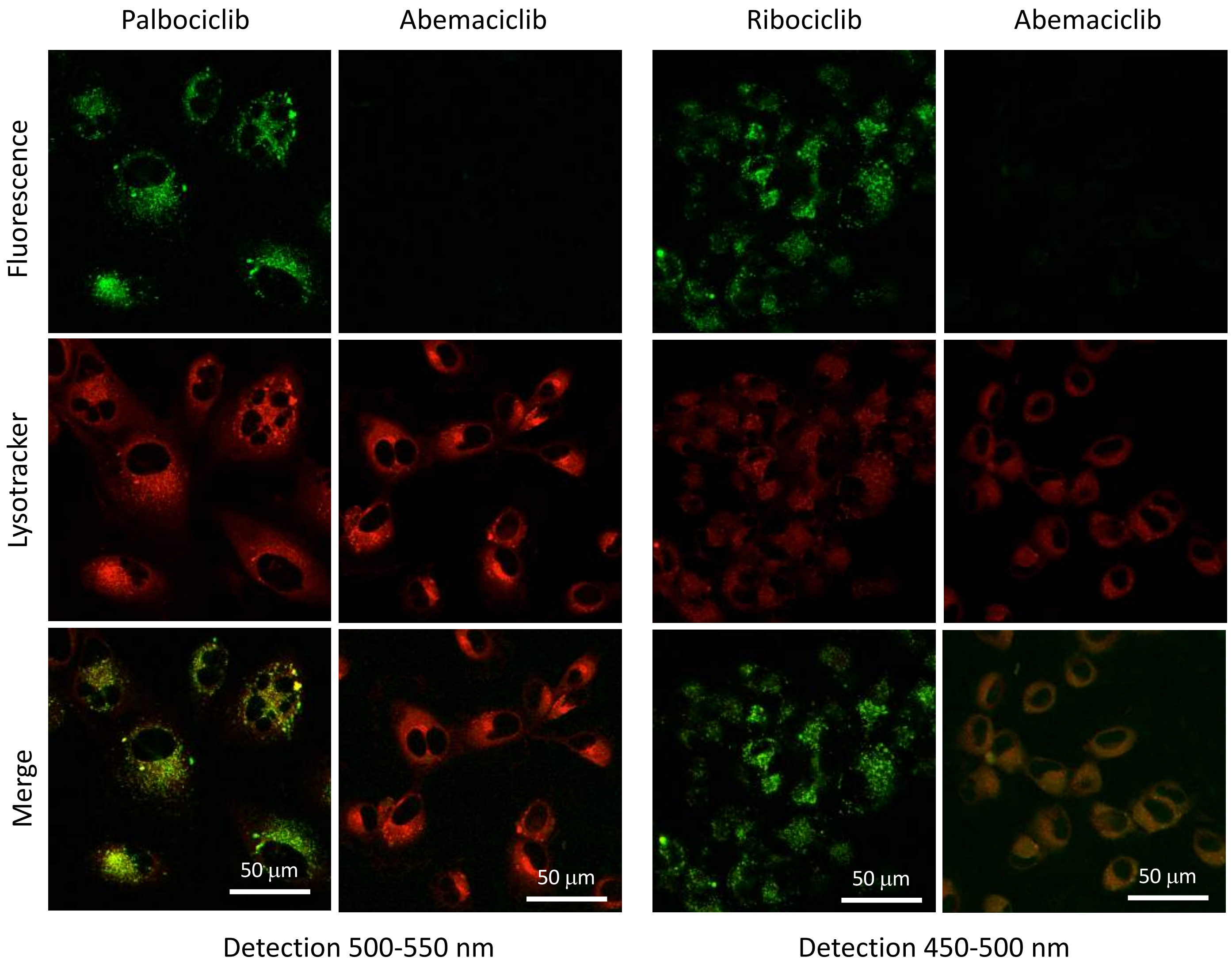
Palbociclib
Abemaciclib
Ribociclib
Abemaciclib
Fluorescence
Lysotracker
Merge
50 µm
50 µm
50 µm
50 µm
Detection 500-550 nm
Detection 450-500 nm

e

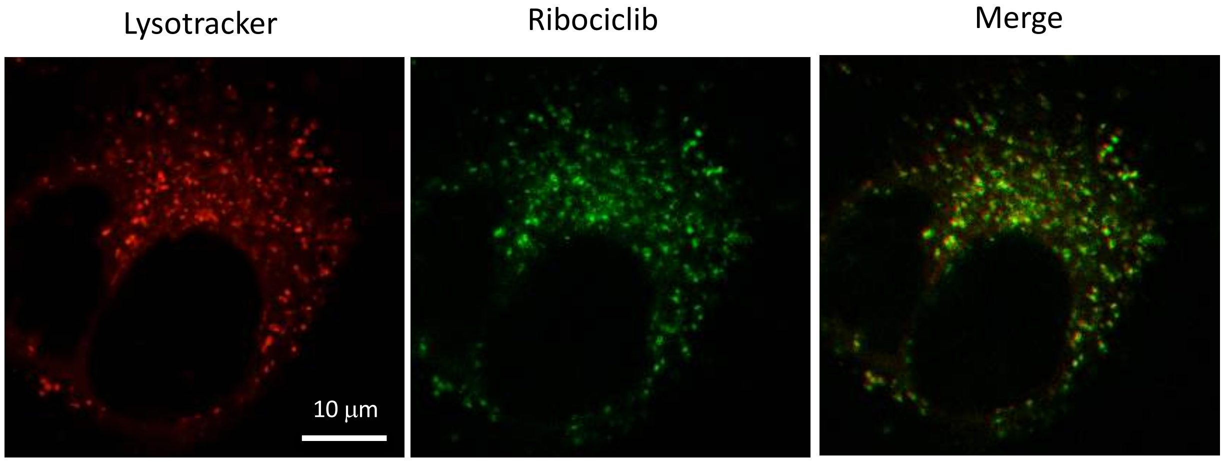
Lysotracker
Ribociclib
Merge
10 µm

Figure S7. (a) Molecular structures of CDK4 inhibitors and their respectives pKa values. (**b**) SA-βgal activity of SK-Mel-103 cells pre-treated with the indicated concentrations of CDK4 inhibitors for 7 days. (**c**) Emission profile of palbociclib, and ribociclib following excitation with a 405 nm laser. The drug was diluted in milli-Q water. Values correspond to one single biological replica. (**d**) Confocal images illustrating the co-localization of lysotracker red and palbociclib (excitation 405 mm/emission 500-550 nm) or ribociclib fluorescence (excitation 405 mm/emission 450-500 nm) in live SK-Mel-103 cells treated with CDK4 inhibitors for 7 days. The drugs were added only once and the media were not changed for the length of the treatment. To improve detection cells were incubated with 4 μM concentration of each drug 1 hour prior image acquisition. Abemaciclib fluorescence was not detected in cells exposed to abemaciclib with the experimental settings applied for the detection of either palbociclib (filter of 500-550 nm) or ribociclib (filter of 450-500 nm). (**e**) Confocal images illustrating the co-localization of lysotracker red and ribociclib-fluorescence (excitation 405 mm/emission 450-500 nm) in live SK-Mel-103 cells exposed to ribociclib 1μM for 7 days. To improve detection cells were incubated with 4 μM ribociclib 1 hour prior image acquisition.
